# Long-range diastereoselectivity in Ugi reactions of 2-substituted dihydrobenzoxazepines

**DOI:** 10.3762/bjoc.7.109

**Published:** 2011-07-13

**Authors:** Luca Banfi, Andrea Basso, Valentina Cerulli, Valeria Rocca, Renata Riva

**Affiliations:** 1 Department of Chemistry and Industrial Chemistry, University of Genova, I-16146 Genova, Italy

**Keywords:** benzoxazepines, cyclic imines, long range stereoinduction, multicomponent reactions, Ugi reaction

## Abstract

The Ugi reaction of 2-substituted dihydrobenzoxazepines was found to proceed with unexpectedly good diastereoselectivitiy (diastereoisomeric ratios up to 9:1), despite the large distance between the pre-existing stereogenic centre and the newly generated one. This result represents the first good 1,4 asymmetric induction in an Ugi reaction as well as the first example of diastereoselective Ugi reaction of seven membered cyclic imines. It allows the diversity-oriented synthesis of various tetrahydro[*f*][1,4]benzoxazepines.

## Introduction

The Ugi reaction is probably the most renowned and widely used multicomponent reaction. Its great utility in the highly convergent and diversity-oriented synthesis of libraries of heterocyclic compounds, stemming from the possibility to introduce up to four diversity inputs in a single step, has been fully demonstrated [1–5]. However, a main drawback of this venerable reaction is the poor diastereoselectivity typically experienced when using chiral inputs. It is well known that chiral isocyanides, aldehydes/ketones and carboxylic acids always bring about no or very little diastereoselectivity, whereas some relative asymmetric induction has been reported only with chiral amines as auxiliaries [6], or with chiral cyclic imines.

The use of cyclic imines (three-component Ugi–Joullié reaction) [7–8] is particularly useful, because the resulting Ugi products are necessarily nitrogen heterocycles. However, good diastereoselectivity has been obtained so far only with a few types of chiral substrates [9–15]. In most cases these are represented by five-membered imines (pyrrolines) with a stereogenic centre α to the imine carbon (1,2-induction), although this relative arrangement is not a guarantee of good stereoselectivity [8,14,16]. Examples of 1,3-induction on chiral imines with the stereocentre β to the carbon [14,16], or α to the nitrogen, of the C=N moiety [13,15,17] are rarer. More often, when the stereocentre is not in α, poor diastereoselectivity is observed [18–19]. This fact limits the diversity of heterocycles that can be accessed stereoselectively from the three-component Ugi–Joullié reaction of cyclic imines.

## Results and Discussion

We report here some preliminary results disclosing a new family of chiral 7-membered cyclic imines that afford good levels of diastereoselectivity when submitted to an Ugi–Joullié reaction, despite the fact that the stereogenic centre is only γ to the imine carbon (1,4 relative induction). This is, to our knowledge, the first example of 1,4 asymmetric induction in an isocyanide based multicomponent reaction of chiral carbonyl compounds or imines, and the first example of diastereoselective Ugi reaction on chiral seven-membered imines [20–21].

**Scheme 1:**
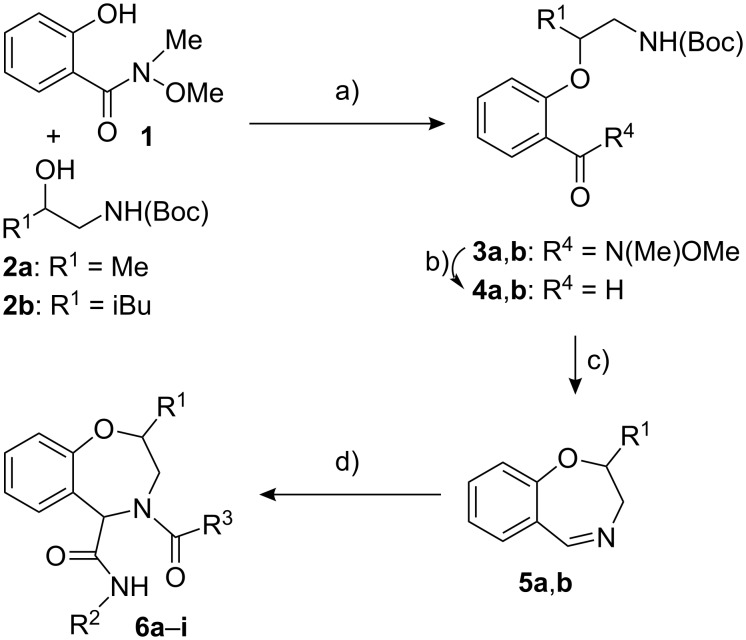
a) TBAD [(*t*-BuO_2_C−N=)_2_], PPh_3_, THF, −15 °C → rt, 49% (**3a**), 62% (**3b**); b) LiAlH_4_, Et_2_O–THF, 0 °C, 90% (**4a**), 88% (**4b**); c) HCl, CH_2_Cl_2_–H_2_O, rt; d) R^2^NC, R^3^CO_2_H, MeOH, rt.

The two imines **5a**,**b** (Scheme 1) have been convergently synthesized in three steps from Weinreb hydroxamate **1**, in turn prepared in one step from salicylic acid (Supporting Information File 1). The key step of the synthesis is the intramolecular condensation of **1** with racemic alcohols **2a**,**b** through a Mitsunobu reaction. The moderate yields are due to the consumption of alcohols **2**, which undergo side-reactions, resulting in incomplete transformation of **1**, even when using 1.3–1.5 equiv of **2**. The use of a larger excess of **2** would probably increase the yields, but this is not particularly convenient (especially if one plans to use enantiomerically pure **2**). In any case, unreacted **1** may be recovered. Alcohol **2b** behaves somewhat better than **2a** in this reaction. The other two steps proceeded with no problems to give imines **5a**,**b** in high yield. It is worth noting that the Mitsunobu reaction is not effective on unprotected salicylaldehyde. 2,3-Dihydrobenzo[*f*][1,4]oxazepines similar to **5a**,**b** have been previously prepared, but through less general routes [22–24].

Compounds **5a**,**b** were reacted with a series of isocyanides and carboxylic acids to give, in good yields, nine different tetrahydro[*f*][1.4]benzoxazepines **6**, equipped with three diversity points.

As shown in Table 1, all the tested Ugi reactions proceeded with remarkably high diastereoselectivity, if one considers that the R^1^ substituent is quite far away from the imine carbon. This long range diastereoselectivity (from 5.25:1 up to 9:1) is completely unprecedented for an isocyanide based multicomponent reaction.

**Table 1:** Results of Ugi reactions of imines **5a**,**b**.

Product	R^1^	R^2^	R^3^	Yield^a^	dr^b^

**6a**	Me	Cy	Et	70%	85:15
**6b**	Me	*t*-Bu	MeOCH_2_	77%	87:13
**6c**	Me	Bn	BocNHCH_2_	71%	84:16
**6d**	iBu	4-BnOC_6_H_4_CH_2_CH_2_	MeOCH_2_	56%	90:10
**6e**	iBu	Cy	Et	59%	86:14
**6f**	iBu	*t*-Bu	Bn	64%	88:12
**6g**	iBu	*n*-Bu	3-BrC_6_H_4_	57%	88:12
**6h**	iBu	*t*-Bu	5-Cl-2-thienyl	78%	89:11
**6i**	iBu	*n*-Bu	Z-NH-CH_2_CH_2_	70%	88:12

^a^Isolated yields (after chromatography) from aldehydes **4a**,**b**. ^b^Determined by HPLC or by ^1^H NMR (for **6f**, **6h**, **6i** only by NMR).

**Figure 1:**
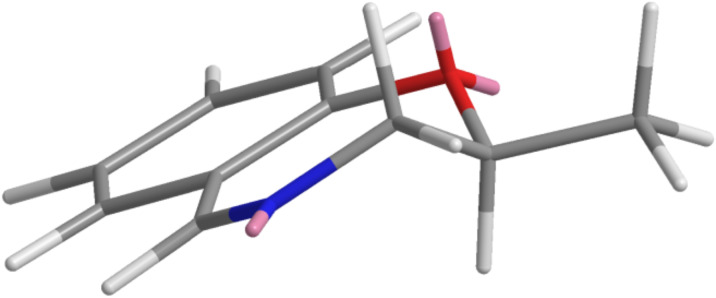
Model of the expected preferred conformation of imine **5a**, as minimized using CSC Chem3D (MOPAC-PM3).

A slight increase in the dr was observed on passing from R^1^ = Me to bulkier R^1^ = iBu. On the other hand the structure and the nature of both isocyanides and carboxylic acids seem to have little influence on the diastereoselectivity. NMR characterization of the products is reported in Supporting Information File 2. Minimization of the cyclic imine **5a** using CSC Chem3D (v10) indicates that there are only two significant conformations, and that the one with the substituent at C-2 in the equatorial position is strongly favored. In this situation (Figure 1), the substituent at C-2 should be quite far away from the site of isocyanide attack, being unable to discriminate the two diastereotopic faces.

**Scheme 2:**
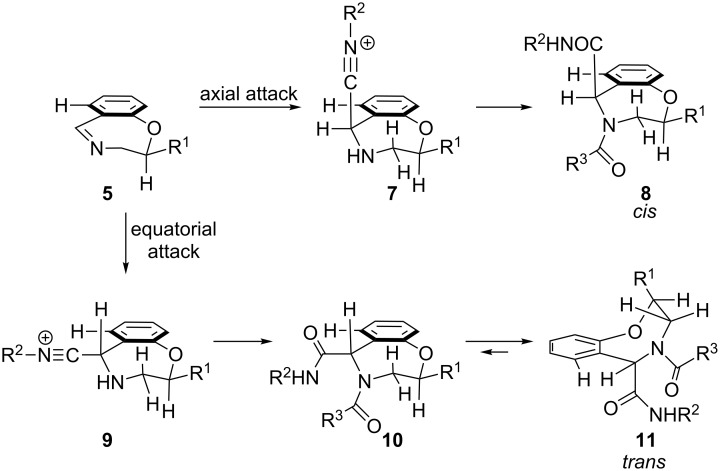
Possible explanation of diastereoselectivity in Ugi reactions of imines **5**.

Thus, rationalization of the observed stereoselectivity is not trivial, also because we have not yet proved unambiguously the relative configuration of the major adducts. Some authors have suggested, for six membered rings, a preferential axial attack of the isocyanide [14,16], since it relieves unfavorable steric strain in the forming tetrahedric adduct (Scheme 2). In our case, equatorial attack, leading to intermediate **9**, would experience steric strain with the *peri* H-7. Therefore, if the preferred conformation of the imine is the one depicted in Figure 1, with R^1^ equatorial, axial attack would give the *cis* adducts. The importance of the unfavorable *peri* interaction is confirmed by the fact that the isocyanide derived substituent prefers an axial position in both stereoisomers, as demonstrated by NOE experiments carried out on **6e** (Supporting Information File 2). Thus, after attack, the *trans* initial adduct **10** undergoes a conformational change to **11**. The two vicinal *J*_2-3_ (i.e., 2.1 and 9.3 Hz for **6h**) in the major diastereomers are in agreement with the chair-like conformation **8** of the *cis* adduct, whereas the same coupling constants in the minor diastereoisomer (i.e., 3.6 and 8.7 Hz. for **6h**) fit the boat-like conformation **11** of the *trans* adduct. However, the difference between these coupling constants for the two stereoisomers is not large enough to guarantee the undisputable assignment of the *cis* relative configuration to the major adduct. In the presented hypothesis, the function of the substituent at C-2 would therefore not be to shield one of the two diastereotopic faces, but only to fix the conformation by favoring an equatorial disposition of R^1^. We are planning to prove the relative configuration of the major adducts and to prepare analogues with further substituents in order to get more clues on this unusual diastereoselectivity and, hopefully, to further improve stereoselectivity.

In conclusion, the methodology presented herein appears particularly well suited for the stereoselective preparation of libraries of peptidomimetics based on the tetrahydrobenzoxazepine ring. Although structures of general formula **6** are unprecedented, other tetrahydrobenzoxazepines have shown interesting pharmacological properties [25–26]. The possibility to introduce up to 4 points of diversity (including also substituents on the aromatic ring), and to obtain enantiomerically pure compounds, starting from enantiomerically pure alcohols **2a**,**b**, will be explored, too.

## Supporting Information

File 1 Complete experimental procedures.

File 2 NMR characterization of products **6** and NMR spectra.

## References

[1] Akritopoulou-Zanze I, Djuric S W (2007). Heterocycles.

[2] Banfi L, Basso A, Riva R, Orru R V A, Ruijter E (2010). Synthesis of Heterocycles via Multicomponent Reactions I.

[3] Banfi L, Riva R, Basso A (2010). Synlett.

[4] Dömling A (2006). Chem Rev.

[5] Hulme C, Dietrich J (2009). Mol Div.

[6] Banfi L, Basso A, Guanti R, Riva R, Zhu J P, Bienaymé H (2005). Asymmetric Isocyanide-Based MCRs. Multicomponent Reactions.

[7] Nutt R F, Joullié M M (1982). J Am Chem Soc.

[8] Bowers M M, Carroll P, Joullié M M (1989). J Chem Soc, Perkin Trans 1.

[9] Znabet A, Ruijter E, de Kanter F J J, Köhler V, Helliwell M, Turner N J, Orru R V A (2010). Angew Chem, Int Ed.

[10] Bonger K M, Wennekes T, Filippov D V, Lodder G, van der Marel G A, Overkleeft H S (2008). Eur J Org Chem.

[11] Chapman T M, Davies I G, Gu B, Block T M, Scopes D I C, Hay P A, Courtney S M, McNeill L A, Schofield C J, Davis B G (2005). J Am Chem Soc.

[12] Timmer M S M, Risseeuw M D P, Verdoes M, Filippov D V, Plaisier J R, van der Marel G A, Overkleeft H S, van Boom J H (2005). Tetrahedron: Asymmetry.

[13] Maison W, Lützen A, Kosten M, Schlemminger I, Westerhoff O, Saak W, Martens J (2000). J Chem Soc, Perkin Trans 1.

[14] Sperger C A, Mayer P, Wanner K T (2009). Tetrahedron.

[15] Zhu D, Chen R, Liang H, Li S, Pan L, Chen X (2010). Synlett.

[16] El Kaïm L, Grimaud L, Oble J, Wagschal S (2009). Tetrahedron Lett.

[17] Gröger H, Hatam M, Martens J (1995). Tetrahedron.

[18] Banfi L, Basso A, Guanti G, Merlo S, Repetto C, Riva R (2008). Tetrahedron.

[19] Iizuka T, Takiguchi S, Kumakura Y, Tsukioka N, Higuchi K, Kawasaki T (2010). Tetrahedron Lett.

[20] Gulevich A V, Shevchenko N F, Balenkova E S, Roeschenthaler G V, Nenajdenko V G (2009). Synlett.

[R21] 21Recently, a very high diastereoselectivity leading to seven-membered ring was obtained in an intramolecular Ugi reaction of an oxoacid endowed with axial chirality: Mehta, V. P.; Modha, S. G.; Ruijter, E.; Van Hecke, K.; Van Meervelt, L.; Pannecouque, C.; Balzarini, J.; Orru, R. V. A.; Van der Eycken, E. *J. Org. Chem.* **2011,** *76,* 2828–2839. doi:10.1021/jo200251q

[22] Del Buttero P, Molteni G, Papagni A, Miozzo L (2004). Tetrahedron: Asymmetry.

[23] Prabhu K R, Sivanand P S, Chandrsekaran S (1998). Synlett.

[24] Takács M, Vámos J, Tóth G, Mikó-Hideg Z (2000). Arch Pharm.

[25] Díaz-Gavilán M, Rodríguez-Serrano F, Gómez-Vidal J A, Marchal J A, Aránega A, Gallo M Á, Espinosa A, Campos J M (2004). Tetrahedron.

[26] Mishra J K, Samanta K, Jain M, Dikshit M, Panda G (2010). Bioorg Med Chem Lett.

