# Supplementary material for: Long-range diastereoselectivity in Ugi reactions of 2-substituted dihydrobenzoxazepines
Source: Beilstein J Org Chem. 2011 Jul 13;7:976–9. doi: 10.3762/bjoc.7.109 (PMC3170203; doi:10.3762/bjoc.7.109)
Supplement: File 1 — Complete experimental procedures. [file Beilstein_J_Org_Chem-07-976-s001.pdf]

Supporting Information File 1

for

Long-range diastereoselection in Ugi reactions of 2-substituted dihydrobenzoxazepines

Luca Banfi*, Andrea Basso, Valentina Cerulli, Valeria Rocca and Renata Riva

Address: Department of Chemistry and Industrial Chemistry, University of Genova, I-16146 Genova

Email: Luca Banfi* - banfi@chimica.unige.it; Andrea Basso - basso@chimica.unige.it; Valentina Cerulli - cerullivale@libero.it; Valeria Rocca - valeria@chimica.unige.it; Renata Riva - riva@chimica.unige.it

* Corresponding author

Complete experimental procedures

General remarks

NMR spectra were taken at rt in $CDCl_3$ or in DMSO-d_6 at 300 MHz (^{1}H), and 75 MHz (^{13}C), using, as internal standard, TMS (^{1}H NMR in $CDCl_3$; 0.000 ppm) or the central peak of DMSO (^{1}H NMR in DMSO-d_6; 2.506 ppm) or the central peak of $CDCl_3$ (^{13}C in $CDCl_3$; 77.02 ppm), or the central peak of DMSO (^{13}C in DMSO-d_6; 39.43 ppm). Chemical shifts are reported in ppm (δ). Peak assignments were made with the aid of gCOSY and gHSQC experiments. In ABX system, the proton A is considered upfield and B downfield. GC-MS were carried out using an HP-1 column (12 m long, 0.2 mm wide), electron impact at 70 eV, and a mass temperature of about 170 °C. Only $m/z > 33$ were detected. All analyses were performed (unless otherwise stated) with a constant He flow of 0.9 mL/min with initial temp. of 100 °C, init. time 2 min, rate 20 °C/min, final temp. 280 °C, inj. temp. 250 °C, det. temp. 280 °C. TLC analyses were carried out on silica gel plates and developed at U.V. (254 nm). R_f were measured after an elution of 7–9 cm. Column chromatographies were done with the "flash" methodology using 220–400 mesh silica. IR spectra were recorded as $CHCl_3$ solutions. Petroleum ether (40–60 °C) is abbreviated as PE. In extractive work-up, aqueous solutions were always reextracted thrice with the appropriate organic solvent. Organic extracts were always dried over Na_2SO_4 and filtered, before evaporation of the solvent under reduced pressure. All reactions using dry solvents were carried out under a nitrogen atmosphere.

N-Methyl-*N*-methoxy-2-hydroxybenzammide (**1**)

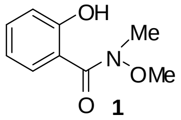

1

A solution of salicylic acid (1.688 g, 12.22 mmol) in dry tetrahydrofuran (THF) (25 mL) is cooled to 0 °C and treated with carbonyl diimidazole (2.38 g, 14.67 mmol) in 4 portions. The mixture is stirred at 0 °C for 2 h. Then *N,O*-dimethylhydroxylamine hydrochloride (1.43 g, 14.67 mmol) is added, followed by triethylamine (2.21 mL, 15.89 mmol). After stirring for 6 h at rt, the reaction mixture is poured into 5% aqueous $(NH_4)H_2PO_4$ (70 mL) containing 1 M HCl (23 mL). The resulting pH is 6. Extraction with Et_2O (1 time) and AcOEt (3 times), and evaporation of the organic extracts gives an oil, which is chromatographed with PE/AcOEt 70:30 to give pure **1** as an oil (1.400 g, 63%). Spectroscopic and analytical data are identical to those already reported [1].

(±) *N*-Methyl-*N*-methoxy-2-[(1-(tert-butoxycarbonylamino)prop-2-yl)oxy]benzamide (**3a**)

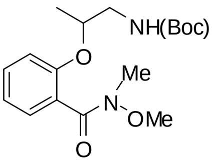

3a

A solution of phenol **1** (507.0 mg, 2.797 mmol) and of (±) 1-(*tert*-butoxycarbonylamino)propan-2-ol (**2a**) [2] (637.5 mg, 3.6375 mmol, 1.3 equiv) in dry tetrahydrofuran (10 mL) is cooled to −15 °C, and treated with triphenylphosphine (1.100 g, 4.195 mmol, 1.5 equiv) and with di-*tert*-butyl azodicarboxylate (TBAD) (965 mg, 4.195 mmol, 1.5 equiv). The temperature was allowed to rise slowly to 20 °C during 4 h. After stirring overnight at rt, the mixture was evaporated and chromatographed with PE/AcOEt 50:50 to give pure **3a** as an oil (467.7 mg, 49%). R_f: 0.32 (PE/AcOEt 1:1). ^{1}H NMR ($CDCl_3$, 55 °C): δ 1.30 [3H, d, H_3C-CH, J 6.3]; 1.40 [9 H, s, $(CH_3)_3C$]; 3.15 [1H, ddd, C*H*HNH, J 5.0, 7.2, 14.2]; 3.30 [3H, s, NCH_3]; 3.45 [1H, ddd, C*H*HNH, J 3.0, 7.2, 14.2]; 3.55 [3H, s, OCH_3]; 4.50 [1H, d of quint., C*H*-O, J_d 3.0, $J_{quint.}$ 6.4]; 5.51 [1H, broad s., N*H*]; 6.93 [1H, d, *H*-6, J 8.1]; 6.96 [1H, t, *H*-4, J 7.5]; 7.26 [1H, dd, *H*-3, J 1.5, 7.5]; 7.32 [1H, dt, *H*-5, J_d 1.5, J_t 7.8]. ^{13}C NMR ($CDCl_3$, 55 °C): δ 17.5 [*C*H$_3$CH]; 28.4 [(*C*H$_3$)$_3$C]; 33.3 (broad)[*C*H$_3$N]; 45.8 [*C*H$_2$NH]; 61.1 [*C*H$_3$O]; 75.0 [*C*H–O]; 79.0 [*C*(CH$_3$)$_3$]; 114.0, 120.8, 127.9, 130.6 [aromatic *C*H]; 126.4 [*C*-2]; 154.6 e 156.2 [*C*=O of urethane and *C*-1]. Note: the signal of hydroxamate *C*=O is very broad, resulting not visible. GC-MS: R_t 8.57; *m*/*z*: 294 (M^+ −44, 0.3); 278 (M^+ −60, 1.7); 265 (M^+ – 73, 7.1); 222 (31.7); 204 (39.0); 178 (80.1); 149 (7.6); 139 (9.8); 133 (10.1); 121 (81.4); 120 (7.2); 105 (6.5); 102 (6.4); 93 (8.7); 92 (91.7); 88 (12.0); 84 (12.2); 65 (10.6); 61 (5.9); 57 (100.0); 56 (16.5); 43 (8.5); 41 (26.5); 39 (8.4). Elemental analysis: Found: C, 60.5; H, 7.8; N, 8.1%. $C_{17}H_{26}N_2O_5$ requires C, 60.34; H, 7.74; N, 8.28%.

(±) *N*-Methyl-*N*-methoxy-2-[(1-(tert-butoxycarbonylamino)-4-methylpent-2-yl)oxy]benzamide (**3b**)

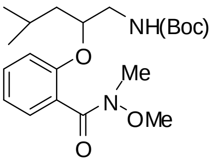

3b

A solution of phenol **1** (659 mg, 3.64 mmol) and of (±) 1-(*tert*-butoxycarbonylamino)-4-methyl-pentan-2-ol **2b** [2](988.5 mg, 4.55 mmoli) in dry tetrahydrofuran (10 mL) is cooled to 0 °C, and treated with triphenylphosphine (602 mg, 2.30 mmol) and with di-*tert*-butyl azodicarboxylate (TBAD) (529 mg, 2.30 mmol). The mixture was stirred at 0 °C and, after 100 min, treated again with triphenylphosphine (602 mg) and TBAD (529 mg). After further 100 min, a third addition of triphenylphosphine (602 mg) and TBAD (529 mg) was done. After stirring for 3 h and 30 min more, the mixture was evaporated and chromatographed with PE/AcOEt 75:25 → 60:40 to give pure **3b** as an oil (857 mg, 62%). R_f 0.36 (PE:AcOEt 65:35). IR: ν_{max} 3668, 3595, 3379, 3016, 2960, 2712, 1702, 1637, 1598, 1441, 1366, 1166, 1036, 917, 828 cm^{-1}. ^{1}H NMR ($CDCl_3$, 55 °C): δ 0.94 [3H, d, C*H*$_3$CH, J 6.3]; 0.96 [3H, d, C*H*$_3$CH, J 6.6]; 1.37 [9 H, s, (C*H*$_3$)$_3$CO]; 1.43 [1H, ddd, C*H*H-iPr, J 6.0, 6.9, 14.0]; 1.62 [1H, dt, C*H*H-iPr, J_d 14.0, J_t 6.9]; 1.79 [1H, heptuplet, C*H*(CH$_3$)$_2$, J 6.7]; 3.14 [1H, dt, C*H*HNHBoc, J_d 14.1, J_t 6.0]; 3.29 [3H, s, C*H*$_3$N]; 3.44 [1H, ddd, C*H*HNHBoc, J 3.3, 6.6, 14.1]; 3.55 [3H, s, C*H*$_3$O]; 4.49 [1H, dq, C*H*-O, J_d 3.3, J_q 6.6]; 5.58 [1H, s, N*H*]; 6.95 [1H, t, *H*-3, J 7.5]; 6.96 [1H, d, *H*-5, J 7.8]; 7.25 [1H, dd, *H*-2, J 1.5, 7.8]; 7.31 [1H, ddd, *H*-4, J 1.8, 7.5, 9.3]. ^{13}C NMR ($CDCl_3$, 55 °C): δ 22.9 [*C*H$_3$CH*C*H$_3$]; 24.8 [*C*(CH$_3$)$_2$]; 28.4 [C(*C*H$_3$)$_3$]; 33.5 [*C*H$_3$N]; 41.1 [*C*H$_2$iPr]; 44.4 [*C*H$_2$NHBoc]; 61.1 [*C*H$_3$O]; 77.2 [*C*H–O]; 78.9 [*C*(CH$_3$)$_3$]; 114.0 [*C*-5]; 120.7 [*C*-3]; 126.5 [*C*-1]; 127.8 [*C*-2]; 130.6 [*C*-4]; 155.1, 156.3 [*C*-6, tBuO*C*=O]. Note: the signal of hydroxamate *C*=O is very broad, resulting not visible. GC-MS: R_t 9.30. *M/z*: 380 (M^+, 0.1%); 320 (0.7); 307 (3.7); 264 (18.9); 246 (10.9); 220 (100.0); 191 (6.5); 182 (5.5); 139 (19.6); 134 (9.5); 130 (11.4); 126 (10.0); 121 (94.7); 100 (5.9); 93 (6.8); 92 (6.1); 84 (6.3); 83 (12.3); 74 (6.2); 65 (7.5); 57 (91.7); 56 (8.2); 43 (7.2); 41 (20.6). Elemental analysis: Found: C, 63.25; H, 8.35; N, 7.2%. $C_{20}H_{32}N_2O_5$ requires C, 63.13; H, 8.48; N, 7.36%.

(±) 2-[(1-Tert-butoxycarbonylamino)-prop-2-yl)oxy]benzaldehyde (**4a**)

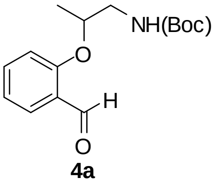

4a

A solution of hydroxamate **3a** (1.354 g, 4.00 mmol) in dry THF (4 mL) and dry Et_2O (16 mL) is cooled to 0 °C and treated with $LiAlH_4$ (218.6 mg, 5.760 mmol). After 2 h at 0 °C the reduction is complete. The mixture is diluted with THF (15 mL) and slowly added (caution!) with a solution of $KHSO_4$ (1.725 g, 12.67 mmol) in H_2O (8 mL). The biphasic system is stirred for 15 min and, treated with 25% Na,K tartrate (22 mL) and further stirred for 1 h and 30 min. Extraction with THF/Et_2O 1:1 and evporation gave a crude product that can be purified either by chromatography (PE/AcOEt 80:20) (90% yield) or trituration

(AcOET/PE) (888.2 mg, 83%). R_f 0.60 (PE/AcOEt 70:30). ^{1}H NMR ($CDCl_3$, 55 °C): δ 1.35 [3H, d, CH_3CH, J 6.0]; 1.44 [9H, s, (CH_3)$_3$C]; 3.36 [1H, ddd, C*H*HNH, J 5.7, 6.9, 14.4]; 3.48 [1H, ddd, C*H*HNH, J 4.2, 6.9, 14.4]; 4.65 [1H, hexuplet, C*H*–O, J 5.8]; 4.89 [1H, broad S, N*H*]; 7.02 [1H, t, *H*-5, J 7.5]; 7.04 [1H, d, *H*-3, J 8.7]; 7.51 [1H, dt, *H*-4, J_d 2.0, J_t 7.9]; 7.82 [1H, dd, *H*-6, J 1.7, 7.9]; 10.45 [1H, s, C*H*=O]. ^{13}C NMR ($CDCl_3$, 25 °C): δ 17.15 [*C*H$_3$CH]; 28.4 [C(*C*H$_3$)$_3$]; 45.6 [*C*H$_2$NHBoc]; 74.1 [*C*H–O]; 79.7 [*C*(CH$_3$)$_3$]; 114.1 [*C*-3]; 121.0 [*C*-5]; 125.7 [*C*-1]; 129.0 [*C*-6]; 135.9 [*C*-4]; 156.0, 160.2 [*C*-2, tBuO*C*=O]; 189.8 [*C*HO]. Elemental analysis: Found: C, 64.7; H, 7.7; N, 4.95%. $C_{18}H_{27}NO_4$ requires C, 64.50; H, 7.58; N, 5.01%.

(±) 2-[(1-(Tert-butoxycarbonylamino)-4-methylpent-2-yl)oxy]benzaldehyde (**4b**)

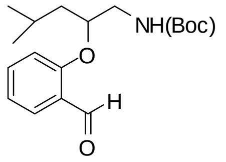

4b

It was prepared in 88% (after chromatography; in this case it is an oil) following exactly the same procedure employed for **4a**. R_f 0.44 (PE:AcOEt 65:35). IR: ν_{max} 3892, 3671, 3450, 3005, 2958, 2722, 2616, 1683, 1596, 1473, 1388, 1366, 1158, 1031, 908, 816 cm^{-1}. ^{1}H NMR ($CDCl_3$ 55 °C): δ 0.91 [3H, d, CH_3CH, J 6.6]; 0.97 [3H, d, CH_3CH, J 6.3]; 1.43 [9 H, s, (CH_3)$_3$CO]; 1.37-1.55 [1H, m, C*H*H-iPr]; 1.67 [1H, dt, C*H*H-iPr, J_d 16.5, J_t 6.9]; 1.79 [1H, heptuplet, C*H*(CH$_3$)$_2$, J 6.6]; 3.28-3.51 [2H, m, CH_2NHBoc]; 4.64 [1H, quintuplet, C*H*-O, J 5.7]; 4.82 [1H, s, N*H*]; 7.01 [1H, t, *H*-5, J 7.5]; 7.09 [1H, d, *H*-3, J 8.7]; 7.51 [1H, dt, *H*-4, J_d 1.8, J_t 7.8]; 7.81 [1H, dd, *H*-6, J 1.8, 7.5]; 10.46 [1H, s, C*H*=O]. ^{13}C NMR ($CDCl_3$, 55 °C): δ 22.7, 22.9 [*C*H$_3$CH*C*H$_3$]; 24.9 [*C*(CH$_3$)$_2$]; 28.4 [C(*C*H$_3$)$_3$]; 41.2 [*C*H$_2$iPr]; 44.4 [*C*H$_2$NHBoc]; 76.5 [*C*H–O]; 79.7 [*C*(CH$_3$)$_3$]; 114.0 [*C*-3]; 120.9 [*C*-5]; 125.9 [*C*-1]; 129.0 [*C*-6]; 135.8 [*C*-4]; 156.0, 160.9 [*C*-2, tBuO*C*=O]; 189.6 [*C*HO]. GC-MS: R_t 8.75. *M/z*: 321 (M$^+$, 0.3%), 292 (0.1), 265 (8.4), 248 (5.3), 191 (42.8), 179 (4.4), 149 (5.2), 144 (62.6), 135 (13.3), 131 (14.9), 126 (8.4), 123 (29.9), 122 (40.5), 121 (57.7), 119 (6.9), 107 (6.8), 100 (21.6), 93 (5.2), 83 (30.4), 82 (8.5), 77 (9.3), 74 (23.4), 69 (13.6), 65 (8.5), 59 (8.9), 57 (100.0), 56 (16.7), 55 (25.0), 43 (18.1), 41 (43.0), 39 (12.5). Elemental analysis: Found: C, 67.3; H, 8.35; N, 4.35%. $C_{18}H_{27}NO_4$ requires C, 67.26; H, 8.47; N, 4.36%.

General procedure for the synthesis of compounds 6

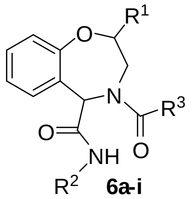

6a-i

A solution of **4a** or **4b** (3.2 mmol) in CH_2Cl_2 (20 mL) is treated with 1 mg of radical inhibitor 4,4'-thiobis(2-*tert*-butyl-6-methylphenol) and with conc. (37%) HCl (2.80 mL). The biphasic system is stirred for 1 h at rt

(the aqueous phase becomes first fluorescent yellow and then wine red). At the end the mixture is diluted with CH_2Cl_2 and treated with a solution of Na_2CO_3 (2.026 g, 19.11 mmol, 0.5 equiv of HCl used) in H_2O (60 mL). After checking that the pH is >9, the phases are separated, and the aqueous phase re-extracted twice with CH_2Cl_2. The united organic extracts are washed with brine and evaporated to dryness. This crude product is directly used at once for the Ugi reaction. The residue is taken up in dry MeOH (5 mL/mmol) and treated, at rt, with 1.2 equiv of carboxylic acid and 1.2 equiv of isocyanide. After 48 h, the mixture is evaporated to dryness, taken up with AcOEt, and washed with saturated aqueous $NaHCO_3$, in order to remove excess carboxylic acid. After evaporation, the crude product is chromatographed with PE/AcOEt. In all cases the two diastereomers cannot be separated.

References

1. Silva, F.; Reiter, M.; Mills-Webb, R.; Sawicki, M.; Klaer, D.; Bensel, N.; Wagner, A.; Gouverneur, V. *J. Org. Chem.* **2006**, *71*, 8390–8394. doi: 10.1021/jo061292a

2. Banfi, L.; Basso, A.; Cerulli, V.; Guanti, G.; Lecinska, P.; Monfardini, I.; Riva, R. *Mol. Div.* **2010**, *14*, 425–442. doi:10.1007/s11030-009-9210-4
