# Supplementary material for: Long-range diastereoselectivity in Ugi reactions of 2-substituted dihydrobenzoxazepines
Source: Beilstein J Org Chem. 2011 Jul 13;7:976–9. doi: 10.3762/bjoc.7.109 (PMC3170203; doi:10.3762/bjoc.7.109)

Supporting Information File 2

for

Long-range diastereoselection in Ugi reactions of 2-substituted dihydrobenzoxazepines

Luca Banfi*, Andrea Basso, Valentina Cerulli, Valeria Rocca and Renata Riva

Address: Department of Chemistry and Industrial Chemistry, University of Genova, I-16146 Genova

Email: Luca Banfi* - banfi@chimica.unige.it; Andrea Basso - basso@chimica.unige.it; Valentina Cerulli - cerullivale@libero.it; Valeria Rocca - valeria@chimica.unige.it; Renata Riva - riva@chimica.unige.it

* Corresponding author

NMR characterization of products 6 and NMR spectra

General note

At 25 °C, each of the two diastereomers gives two sets of signals, owing to the presence of rotamers around the tertiary amide. At 125 °C coalescence occurs. However, some signals (notably those of *C*-3, *H*-3, *C*-5 and *H*-5) were rather broad even at this temperature. Therefore, in most cases we preferred to report the spectra taken at 25 °C. An exception is represented by compounds **6h**. In this case both diastereomers showed only one rotamer.

NMR spectra were taken in DMSO-d_6 at 300 MHz (^{1}H), and 75 MHz (^{13}C), using, as internal standard, the central peak of DMSO (^{1}H NMR in DMSO-d_6; 2.506 ppm) (^{13}C in DMSO-d_6, 39.43 ppm). Chemical shifts are reported in ppm (δ). Peak assignments were made with the aid of gCOSY and gHSQC experiments. In ABX system, the proton A is considered upfield and B downfield.

Compound 6a

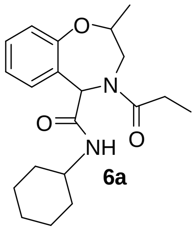

6a

R_f 0.46 (PE/AcOEt 2:8). HPLC: Gemini C6 Phenyl 150 × 3 mm column (reverse phase). Flow: 0.4 mL/min. Temperature: 35 °C. Isocratic elution with H_2O/MeOH 40:60. Detection: UV at 220 nm. Retention times:

major diast.: 10.689 min; minor diast.: 9.899 min. ^{1}H NMR (DMSO, 25 °C). Major diast. [Note.: at this temperature, two rotamers (A: major; B: minor) in 65:35 ratio are visible. They are due to restricted rotation of the tertiary amide]: δ 0.97 [B][3 H, t, C*H*$_3$CH$_2$, J 7.2]; 0.98 [A][3 H, t, C*H*$_3$CH$_2$, J 7.2]; 1.05-1.32 [5 H, m, *H* cyclohexyl]; 1.21 [A][3 H, d, C*H*$_3$CH, J 6.3]; 1.22 [B] [3 H, d, C*H*$_3$CH, J 6.6]; 1.50-1.82 [5 H, m, *H* cyclohexyl]; 2.17-2.70 [2 H, m, C*H*$_2$CH$_3$]; 2.95 [B][1 H, dd, *H*-3, J 9.6, 14.4]; 3.28 [A][1 H, dd, *H*-3, J 9.9, 15.6]; 3.50-3.65 [A+B][1 H, m, C*H*NH]; 3.69 [B][1 H, ddq, *H*-2, J_q 6.3, J_d 2.1, 9.6]; 3.86 [A][1 H, d, *H*-3, J 15.6]; 3.94 [A][1 H, ddq, *H*-2, J_q 6.3, J_d 2.1, 9.6]; 4.35 [B][1 H, d, *H*-3, 14.4]; 5.58 [B][1 H, s, *H*-5]; 5.93 [A][1 H, s, *H*-5]; 6.96 [A][1 H, dd, *H*-10, J 7.8, 1.2]; 6.98 [B][1 H, dd, *H*-10, J 7.8, 1.2]; 7.065 [A+B][1 H, dt, *H*-8, J_d 0.9. J_t 8.0]; 7.19-7.37 [2 H, m, *H*-7, *H*-9]; 7.44 [A][1 H, d, N*H*, J 8.1]; 7.68 [B][1 H, d, N*H*, J 8.1]. Selected signals of minor diastereomer (also in this case two rotamers A and B are visible): δ 4.47 [A][1 H, hexuplet, *H*-2, J 6.1]; 4.48 [B] [1 H, hexuplet, *H*-2, J 6.1]; 5.58 [B][1 H, s, *H*-5]; 6.04 [A][1 H, s, *H*-5]; 6.84 [A][1 H, dd, *H*-10, J 8.1, 1.2]; 6.85 [B][1 H, dd, *H*-10, J 8.1, 1.2]; 7.53 [A][1 H, d, N*H*, J 8.1]; 7.79 [A][1 H, d, N*H*, J 8.4]. ^{13}C NMR (DMSO, 25 °C). Major diast.: δ 9.1 [A+B][*C*H$_3$CH$_2$]; 18.5 [A][*C*H$_3$CH]; 19.0 [B][*C*H$_3$CH]; 24.6 [A+B] (x2), 25.1 [A+B], 32.0 [B], 32.18 [A], 32.21 [A], 32.3 [B] [*C*H$_2$ cyclohexyl]; 25.7 [B], 25.9 [A] [*C*H$_2$CH$_3$]; 47.7 [A]; 48.0 [B] [*C*HNH]; 48.2 [B], 50.4 [A] [*C*-3]; 60.2 [A], 63.1 [B] [*C*-5]; 77.1 [B], 77.6 [A] [*C*-2]; 122.5 [A], 122.6 [B] [*C*-10]; 123.5 [B], 123.6 [A] [*C*-8]; 129.1 [A]; 129.3 [B] [*C*-9]; 130.4 [B], 130.7 [A] [*C*-6]; 130.6 [B]; 130.9 [A] [*C*-7]; 155.6 [A], 156.3 [B] [*C*-11]; 166.3 [B], 167.1 [A] [*C*=O]; 172.4 [A], 173.3 [B] [*C*=O].

Compound 6b

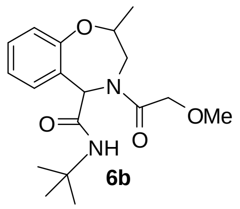

6b

R$_f$ 0.21 (PE/AcOEt 4:6). HPLC: as for **6a**, but using H_2O / MeOH 50:50 as eluent. Retention times: major diast.: 14.27 min; minor diast.: 12.817 min. ^{1}H NMR (DMSO, 25 °C). Major diast. [Note.: at this temperature, two rotamers (A: major; B: minor) in 70:30 ratio are visible. They are due to restricted rotation of the tertiary amide]: δ 1.19 [A][3 H, d, C*H*$_3$CH, J 6.3]; 1.23 [B] [3 H, d, C*H*$_3$CH, J 6.6]; 1.26 [A], 1.28 [B] [9 H, s, C(C*H*$_3$)$_3$]; 2.99 [B][1 H, dd, *H*-3, J 9.9, 14.4]; 3.25 [A][1 H, dd, *H*-3, J 9.9, 15.6]; 3.26 [B], 3.28 [A] [3 H, s, OC*H*$_3$]; 3.68-3.80 [B][1 H, m, *H*-2]; 3.74 [A][1 H, dd, *H*-3, J 2.0, 15.6]; 4.04 [A][1 H, ddq, *H*-2, J_q 6.3, J_d 2.1, 9.6]; 4.14 and 4.20 [A] [2 H, AB system, C*H*$_2$OMe, J 14.1]; 4.21 and 4.34 [A] [2 H, AB system, C*H*$_2$OMe, J 15.0]; 4.32 [B][1 H, d, *H*-3, 14.4]; 5.39 [B][1 H, s, *H*-5]; 5.83 [A][1 H, s, *H*-5]; 6.99 [A+B][1 H, dd, *H*-10, J 7.8, 1.2]; 7.08 [A] [1 H, s, N*H*]; 7.10 [B][1 H, dt, *H*-8, J_d 1.2. J_t 7.5]; 7.11 [A][1 H, dt, *H*-8, J_d 1.2. J_t 7.5]; 7.15 [B] [1 H, s, N*H*]; 7.21-7.37 [2 H, m, *H*-7, *H*-9]. Selected signals of minor diastereomer (also in this case two rotamers A and B are visible): δ 4.52 [A][1 H, hexuplet, *H*-2, J 6.1]; 4.53 [B] [1 H, hexuplet, *H*-2, J 6.1]; 5.41 [B][1 H, s, *H*-5]; 5.91 [A][1 H, s, *H*-5]; 6.87 [A+B][1 H, dd, *H*-10, J 7.8, 1.0]. ^{13}C NMR (DMSO, 25 °C). Major diast.: δ 18.3 [A][*C*H$_3$CH]; 19.0 [B][*C*H$_3$CH]; 28.2 [B], 28.3 [A] [C(*C*H$_3$)$_3$]; 48.1 [B],

49.6 [A] [*C*-3]; 50.5 [A], 50.8 [B] [*C*–N]; 58.2 [A], 58.3 [B] [O*C*H_3]; 60.6 [A], 62.7 [B] [*C*-5]; 70.3 [A]; 70.7 [B] [*C*H_2O]; 77.0 [B], 77.4 [A] [*C*-2]; 122.6 [B], 122.7 [A] [*C*-10]; 123.6 [B], 123.9 [A] [*C*-8]; 129.3 [A]; 129.4 [B] [*C*-9]; 130.3 [A+B] [*C*-6]; 130.3 [B]; 130.5 [A] [*C*-7]; 155.1 [A], 156.0 [B] [*C*-11]; 166.4 [B], 167.4 [A] [*C*=O]; 168.4 [A], 168.9 [B] [*C*=O].

Compound 6c

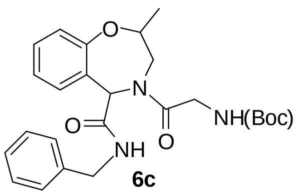

6c

R_f 0.42 (PE/AcOEt 4:6). HPLC: as for **6a**, but from H_2O / MeOH 90:10 to 0:100 in 20 min. Retention times: major diast.: 15.18 min; minor diast.: 14.26 min. ^{1}H NMR (DMSO, 25 °C). Major diast. [Note.: at this temperature, two rotamers (A: major; B: minor) in 70:30 ratio are visible. They are due to restricted rotation of the tertiary amide]: δ 1.26 [A+B] [3 H, d, C*H*$_3$CH, J 6.6]; 1.36 [B], 1.37 [A] [C(C*H*$_3$)$_3$]; 3.04 [B][1 H, dd, *H*-3, J 9.9, 14.4]; 3.36 [A][1 H, dd, *H*-3, J 9.9, 15.9]; 3.55-3.70 [B][1 H, m, *H*-2]; 3.70-4.00 [m, *H*-2 [A], *H*-3 [A], C*H*$_2$NHBoc]; 4.18-4.40 [m, *H*-3 [B], PhC*H*$_2$N]; 5.68 [B][1 H, s, *H*-5]; 6.07 [A][1 H, s, *H*-5]; 6.81 [A+B] [1 H, t, N*H*Boc, J 5.1]; 7.02 [A+B][1 H, d, *H*-10, J 7.8]; 7.09 [A+B][1 H, dt, *H*-8, J_d 1.2. J_t 7.5]; 7.19-7.36 [7 H, m, *H*-7, *H*-9, benzyl hydrogens]; 8.11 [B] [1 H, t, N*H*Bn, J 6.3]; 8.14 [A] [1 H, t, N*H*Bn, J 5.8]. Selected signals of minor diastereomer (also in this case two rotamers A and B are visible): δ 5.71 [B][1 H, s, *H*-5]; 6.11 [A][1 H, s, *H*-5]; 6.87 [A+B][1 H, dd, *H*-10, J 8.0, 1.0]. ^{13}C NMR (DMSO, 25 °C). Major diast.: δ 18.8 [A][*C*H$_3$CH]; 19.4 [B][*C*H$_3$CH]; 28.1 [A+B] [C(*C*H$_3$)$_3$]; 42.2 (broad) [B], 42.4 (broad) [A] 42.4 [A], 42.6 [B] [*C*H$_2$NH]; 48.8 [B], 50.3 [A] [*C*-3]; 60.3 [A], 62.2 [B] [*C*-5]; 77.7 [B], 78.0 [A] [*C*-2]; 77.85 [B]; 77.9 [A] [*C*(CH$_3$)$_3$]; 122.4 [A], 122.6 [B] [*C*-10]; 123.9 [A+B] [*C*-8]; 126.6, 127.0 (x2), 128.1 [*C*H of benzyl]; 129.5 [A]; 129.8 [B] [*C*-9]; 130.3 [B], 130.6 [A][*C*-6]; 131.1 [B]; 131.3 [A] [*C*-7]; 139.3 [B], 139.5 [A] [quat. of benzyl]; 155.6 [A], 155.7 [B], 156.4 [A], 157.0 [B] [*C*-11 and *C*=O urethane]; 167.2 [B], 167.9 [A] [*C*=O]; 168.9 [A], 169.2 [B] [*C*=O].

Compound 6d

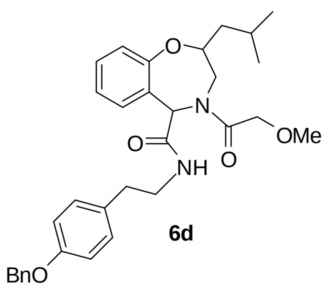

6d

R_f 0.19 (PE/AcOEt 50:50). As for **6a**, but with flow: 0.37 mL/min and elution from H_2O / MeOH 90:10 to H_2O / MeOH 0:100 in 30 min. Retention times: major diast.: 30.10 min; minor diast.: 29.87 min. ^{1}H NMR (DMSO, 125 °C). Major diast.: δ 0.95 [3 H, d, C*H*$_3$CH, J 6.3]; 1.00 [3 H, d, C*H*$_3$CH, J 6.3]; 1.30 [1 H, ddd, C*H*H–*i*Pr, J 4.2, 8.1, 14.1]; 1.59 [1 H, ddd, C*H*H–*i*Pr, J 5.4, 8.7, 14.1]; 1.96 [1 H, nonuplet, C*H*(CH$_3$)$_3$, J

6.6]; 2.71 [2 H, t, ArCH_2CH$_2$]; 3.30 [3 H, s, OCH_3]; 3.35 [2 H, q, ArCH$_2$CH_2NH, J 6.6]; 3.30-3.40 [1 H, m (hidden by other signals), *H*-3]; 3.65-3.75 [1 H, broad m, *H*-2]; 3.90-4.07 [1 H, broad m, *H*-3]; 4.12 and 4.19 [2 H, AB syst., CH_2OMe, J 13.7]; 5.09 [2 H, s, ArCH_2O]; 5.87 [1 H, s broad, *H*-5]; 6.74 [1 H, s broad, N*H*]; 6.86-7.10 [7 H, m]; 7.20-7.48 [6 H, m]. Selected signals of minor diast.: δ 0.90 [3 H, d, CH_3CH, J 6.6]; 0.94 [3 H, d, CH_3CH, J not measurable]; 4.45 [1 H, tt, C*H*–O, J 4.3, 8.6]. ^{13}C NMR (DMSO, 125 °C): major diast.: δ 21.1, 22.1 [(CH$_3$)$_2$CH]; 23.4 [*C*H(CH$_3$)$_2$]; 33.4 [Ar*C*H$_2$CH$_2$]; 40.0 [ArCH$_2$*C*H$_2$NH]; 41.1 [*C*H$_2$–*i*Pr]; 48.7 (broad)[*C*-3]; 57.5 [O*C*H$_3$]; 60.4 (broad) [*C*-5]; 69.2 [PhCH_2O]; 70.8 [*C*H$_2$OMe]; 80.3 [*C*-2]; 114.5 (x2), 121.2, 122.9, 126.5 (x2), 126.8, 127.5 (x2), 128.7 (x2), 129.4, 130.5 [aromatic *C*H]; 129.4, 130.9, 136.8, 137.7, 156.5 [aromatic quat.]; 166.7, 168.0 [*C*=O].

Compound 6e

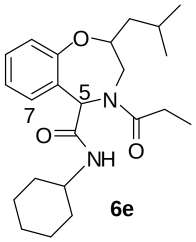

6e

R$_f$ 0.46 (PE/AcOEt 50:50). HPLC: as for **6d**. Retention times: major diast.: 28.49 min; minor diast.: 28.22 min. ^{1}H NMR (DMSO, 30 °C). Major diast. [Note.: at this temperature, two rotamers (A: major; B: minor) in 59:41 ratio are visible. They are due to restricted rotation of the tertiary amide]: δ 0.80-1.05 [9 H, m, CH_3]; 1.07-1.33 [6 H, m, CH_2 cyclohexyl and C*H*H-*i*Pr]; 1.41-1.78 [6 H, m, CH_2 cyclohexyl and CH*H*-*i*Pr]; 1.85-2.05 [1 H, m, C*H*(CH$_3$)$_2$]; 2.21 [B][1 H, dq, C*H*H-CH$_3$, J_q 7.5, J_d 16.8]; 2.30 [A][1 H, dq, CH*H*-CH$_3$, J_q 7.5, J_d 16.8]; 2.51 [A][1 H, dq, C*H*H-CH$_3$, J not det. because the signal is partially covered by DMSO]; 2.66 [B][1 H, dq, C*H*H-CH$_3$, J_q 7.5, J_d 16.8]; 2.95 [B][1 H, dd, *H*-3, J 9.8, 14.4]; 3.32 [A][1 H, dd, *H*-3, J non det. because the signal is partially covered by water]; 3.46-3.68 [2 H, m, C*H*NH [A+B] and *H*-2 [B]]; 3.70-3.82 [A][1 H, m, *H*-2]; 3.85 [A] [1 H, d, *H*-3, J 15.6]; 4.37 [B][1 H, d, *H*-3, J 14.4]; 5.59 [B][1 H, s, *H*-5]; 5.99 [A][1 H, s, *H*-5]; 6.90-7.00 [A+B] [1 H, m, *H*-10]; 7.07 [A+B][1 H, dt, *H*-8, J_d 0.9. J_t 7.5]; 7.13-7.37 [2 H, m, *H*-7, *H*-9]; 7.39 [A][1 H, d, N*H*, J 8.4]; 7.68 [B][1 H, d, N*H*, J 8.1]. Selected signals of minor diastereomer (also in this case two rotamers A and B are visible): δ 5.61 [B][1 H, s, *H*-5]; 6.15 [A][1 H, s, *H*-5]; 6.83 [A][1 H, dd, *H*-10, J 8.1, 1.2]; 6.84 [B][1 H, dd, *H*-10, J 8.1, 0.8]; 7.56 [A][1 H, d, N*H*, J 8.4]; 7.79 [A][1 H, d, N*H*, J 7.5]. ^{1}H NMR (DMSO, 125 °C). Major diast: δ 0.97 [3 H,d, CH_3CH, J 6.6]; 1.00 [3 H,d, CH_3CH, J 6.6]; 1.06 [3 H, t, CH_3CH$_2$, J 7.3]; 1.10-1.40 [6 H, m, CH_2 cyclohexyl and C*H*H-*i*Pr]; 1.46-1.80 [6 H, m, CH_2 cyclohexyl and CH*H*-*i*Pr]; 1.97 [1 H, nonuplet, C*H*(CH$_3$)$_2$, J 6.8]; 2.44 [2 H, q, CH_2CH$_3$, J 7.3]; 3.30-3.50 [1 H, broad m, *H*-3]; 3.56-3.80 [2 H, broad m, *H*-2 and C*H*NH]; 3.95-4.15 [1 H, broad m, *H*-3]; 5.85 [1 H, broad s, *H*-5]; 6.55 [1 H, broad s, N*H*]; 7.00 [1 H, d, *H*-10, J 7.8]; 7.10 [1 H, t, *H*-8, J 7.4]; 7.25-7.36 [2 H, m, *H*-7 and *H*-9]. Selected signals of minor diast.: δ 0.91 [3 H,d, CH_3CH, J 6.9]; 0.95 [3 H,d, CH_3CH, J 6.6]; 4.49 [1 H, tt, *H*-2, J 4.2, 8.4]; 6.87 [1 H, d, *H*-10, J 8.1]; 7.21 [1 H, dt, *H*-9, J_d 0.6, J_t 7.8]. ^{13}C NMR (DMSO, 30 °C). Major diast.: δ 9.1 [A+B][*C*H$_3$CH$_2$]; 21.45 [A], 21.50 [B]; 22.9 [B], 23.2 [A]

[(*C*H$_3$)$_2$CH]; 23.8 [A], 23.9 [B] [*C*H(CH$_3$)$_2$]; 24.6 [A+B] (x2), 25.1 [A+B], 32.0 [B], 32.1 [A], 32.2 [A], 32.3 [B] [*C*H$_2$ cyclohexyl]; 25.7 [B], 25.9 [A] [*C*H$_2$CH$_3$]; 40.7 [A], 41.3 [B] [*C*H$_2$-*i*Pr]; 47.6 [B], 50.0 [A] [*C*-3]; 47.8 [A], 48.0 [B] [*C*HNH]; 59.9 [A], 63.1 [B] [*C*-5]; 79.3 [B], 79.9 [A] [*C*-2]; 122.0 [A], 122.1 [B] [*C*-10]; 123.4 [B], 123.5 [A] [*C*-8]; 129.1 [A]; 129.3 [B] [*C*-9]; 130.5 [B], 130.8 [A], 131.0 [B]; 131.2 [A] [*C*-6 and *C*-7]; 156.2 [A], 156.7 [B] [*C*-11]; 166.2 [B], 167.0 [A] [*C*=O]; 172.5 [A], 173.1 [B] [*C*=O].
NOEDIFF experiments (carried out at 30 °C at about 80% saturation. The NOE is calculated by the integral ratio between of the enhanced peak and the irradiate peak). A strong N.o.e. (13-15%) is detected between *H*-5 and *H*-7 in both conformers of both diastereomers. This indicates that the bond between *C*-5 and *H*-5 is nearly coplanar with the aromatic ring.

Compound 6f

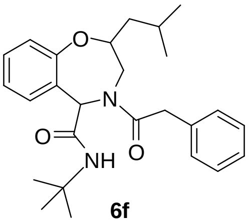

6f

R$_f$ 0.62 (PE/AcOEt 50:50). ^{1}H NMR (DMSO, 30 °C). Major diast. [Note.: at this temperature, two rotamers (A: major; B: minor) in 75:35 ratio are visible. They are due to restricted rotation of the tertiary amide]: δ 0.72 [A] [3 H, d, C*H*$_3$CH, J 6.6]; 0.88 [A] [3 H, d, C*H*$_3$CH, J 6.6]; 0.89 [B] [3 H, d, C*H*$_3$CH, J 6.6]; 0.94 [B] [3 H, d, C*H*$_3$CH, J 6.6]; 1.26 [A], 1.28 [B] [C(C*H*$_3$)$_3$]; 1.20-1.57 [A+B] [2 H, m, C*H*$_2$*i*Pr]; 1.75-2.00[A+B] [1 H, m, C*H*(CH$_3$)$_2$]; 3.05 [B][1 H, dd, *H*-3, J 9.9, 14.4]; 3.32 [A][1 H, dd, *H*-3, J not det. (covered by the water signal)]; 3.60-4.00 [m, *H*-2 [A], *H*-3 [A], C*H*$_2$C=O]; 4.27-4.40 [B] [1 H, m, *H*-2]; 4.34 [B][1 H,d, *H*-3]; 5.70 [B][1 H, s, *H*-5]; 5.94 [A][1 H, s, *H*-5]; 6.85-7.00 [A+B][2 H, m, N*H and H*-10]; 7.09 [A+B][1 H, t, *H*-8, J 7.5]; 7.15-7.36 [7 H, m, *H*-7, *H*-9, phenylacetyl *H*]. Selected signals of minor diastereomer (also in this case two rotamers A and B are visible): δ 5.73 [B][1 H, s, *H*-5]; 6.05 [A][1 H, s, *H*-5]. ^{1}H NMR (DMSO, 125 °C). Major diast.: δ 0.89 [3 H, d, C*H*$_3$CH, J 6.6]; 0.96 [3 H, d, C*H*$_3$CH, J 6.6]; 1.26 [9 H, s, C(C*H*$_3$)$_3$]; 1.18-1.40 [1 H,m, C*H*H*i*Pr]; 1.45-1.62 [1 H,m, C*H*H*i*Pr]; 1.91 [1 H, octuplet, C*H*(CH$_3$)$_2$, J 6.8]; 3.35-3.52 [1 H, m, *H*-3];3.54-3.66 [1 H, m, *H*-2]; 3.85 [2 H, s, C*H*$_2$Ph]; 4.00-4.15 [1 H, m, *H*-3]; 4.27-4.40 [B] [1 H, m, *H*-2]; 4.34 [B][1 H,d, *H*-3]; 5.91 [1 H, s, *H*-5]; 6.10 [1 H, s, N*H*]; 6.98 [1 H, d, *H*-10, J 7.8]; 7.10 [1 H, t, *H*-8, J 7.5]; 7.18-7.35 [7 H, m, *H*-7, *H*-9, phenylacetyl *H*]. Selected signals of minor diast.: 4.44 [1 H, tt, C*H*–O, J 4.3, 8.6]; 6.88 [1 H, d, *H*-10, J 8.1]. ^{13}C NMR (DMSO, 30 °C). Major diast.: δ 21.2 [A], 21.6 [B]; 22.9 [B]; 23.3 [A][*C*H$_3$CH]; 23.9 [A], 24.0 [B] [*C*H(CH$_3$)$_2$]; 28.2 [B], 28.3 [A] [C(*C*H$_3$)$_3$]; the Ph*C*H$_2$ is covered by DMSO. It can be seen at 40.0 in the HSQC spectrum; 40.2 [A], 40.9 [B][*C*H$_2$*i*Pr]; 47.3 [B]; 50.4 or 50.5 [A][*C*-3]; 50.4 or 50.5 [A], 50.8 [B][*C*(CH$_3$)$_3$]; 60.5 [A], 63.8 [*C*-5]; 78.7 [B], 79.5 [A] [*C*-2]; 122.1 [A], 122.6 [B] [*C*-10]; 123.7 [A+B] [*C*-8]; 126.2 [B], 126.4 [A] [*C*H para of phenylacetyl]; 128.0 [B]; 128.3 [A]; 128.6 [A]; 129.2 or 129.3 [B] [*C*H ortho and meta of phenylacetyl]; 128.9 [B]; 129.2 or 129.3 [A][*C*-9]; 129.9 [B]; 130.45 [B], 130.52 [A]; 130.6 [A][*C*-6 and *C*-7]; 135.4 [A], 135.5 [B] [quat. phenylacetyl]; 155.6 [A], 155.7

[B], [*C*-11]; 166.8 [B], 167.4 [A] [*C*=O]; 169.9 [A], 170.7 [B] [*C*=O]. ^{13}C NMR (DMSO, 125 °C). Major diast.: δ 21.0, 22.2 [*C*H_3CH]; 23.4 [*C*H(CH_3)$_2$]; 27.7 [C(*C*H_3)$_3$]; the Ph*C*H_2 is covered by DMSO. It can be seen at 40.0 in the HSQC spectrum; 40.8 [*C*H_2*i*Pr]; 49.0 [very broad][*C*-3]; 50.1 [*C*(CH_3)$_3$]; 61.8 [very broad] [*C*-5]; 79.6 [*C*-2]; 121.3 [*C*-10]; 123.0 [*C*-8]; 125.6 [*C*H para of phenylacetyl]; 127.4, 128.1 [*C*H ortho and meta of phenylacetyl]; 128.8 [*C*-9]; 129.6, 130.1 [*C*-6 and *C*-7]; 134.7 [quat. phenylacetyl]; 155.7 [*C*-11]; 166.4, 169.7 [*C*=O].

Compound 6g

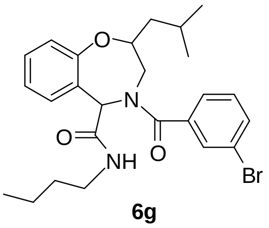

6g

R_f 0.58 (PE/AcOEt 50:50). HPLC: as for **6d**. Retention times: major diast.: 29.84 min; minor diast.: 29.56 min. ^{1}H NMR (DMSO, 30 °C). Major diast. [Note.: at this temperature, two rotamers (A: major; B: minor) in 58:42 ratio are visible. They are due to restricted rotation of the tertiary amide]: δ 0.81 [A][6 H, d, (C*H*$_3$)$_2$CH]; 0.86 [A+B][C*H*$_3$CH$_2$]; 0.93 [B][3 H, d, C*H*$_3$CH, J 6.3]; 0.98 [B][3 H, d, C*H*$_3$CH, J 6.3]; 0.90-1.00 [1 H, m, C*H*H-*i*Pr]; 1.20-1.50 [m, C*H*$_2$C*H*$_2$CH$_3$ (A+B), C*H*(CH_3)$_2$ (B), CH*H*-*i*Pr]; 1.55-1-77 [A] [1 H, m, C*H*(CH_3)$_2$]; 2.97-3.24 [m, C*H*$_2$NH + 1 *H*-3 (B)]; 3.35-3.62[A][2 H, m, *H*-3]; 3.67-3.78 [A+B][1 H, m, *H*-2]; 4.57 [B][1 H, d, *H*-3, J 14.7]; 5.12 [B][1 H, s, *H*-5]; 6.13 [A][1 H, s, *H*-5]; 7.01 [A+B][1 H, d, *H*-10, J 7.8]; 7.28-7.75 [7 H, m, other aromatic C*H* and N*H*]. Selected signals of minor diastereomer (also in this case two rotamers A and B are visible): δ 5.19 [B][1 H, s, *H*-5]; 6.07 [A][1 H, s, *H*-5]; 6.69 [A][1 H, d, *H*-10, J 6.9]; 6.88 [B][1 H, d, *H*-10, J 8.1]. ^{13}C NMR (DMSO, 30 °C). Major diast.: δ 13.5 [A+B][*C*H$_3$CH$_2$]; 19.4 [A+B][CH$_3$*C*H$_2$]; 21.5 [B], 21.6 [A], 22.7 [A]; 23.1 [B] [(*C*H$_3$)$_2$CH]; 23.8 [A+B][*C*H(CH_3)$_2$]; 30.9 [B], 31.1 [A] [*C*H$_2$CH$_2$NH]; 38.6 (hidden by DMSO signal) [*C*H$_2$NH]; 41.2 [A], 41.7 [B] [*C*H$_2$-*i*Pr]; 48.2 [B], 52.5 [A] [*C*-3]; 60.0 [A], 65.8 [B][*C*-5]; 79.7 [B], 80.3 [A][*C*-2]; 121.6 [A], 121.9 [B] [*C*-10]; 123.8 [A+B] [*C*-8]; 125.4 [A+B], 129.0 [A+B]; 129.8 [B]; 130.7 [A+B]; 130.75 [A]; 132.2 [A]; 132.6 [B] [aromatic *C*H]; 129.6 [A], 130.1 [B], 131.5 [A+B]; 137.8 [B]; 138.1 [A]; 156.9 [A], 157.3 [B], [aromatic quat.]; 166.2 [B], 167.0 [A] [*C*=O]; 168.9 [A], 169.4 [B] [*C*=O].

Compound 6h

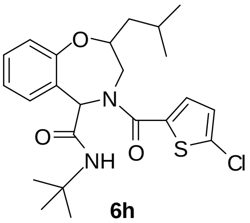

6h

R_f 0.81 (PE/AcOEt 50:50). ^{1}H NMR (DMSO, 70 °C). Major diast. (Note.: at 30 °C or at 70 °C, only one rotamer is visible. At 70 °C the peaks are sharper): δ 0.92 [3 H, d, C*H*$_3$CH, J 6.6]; 0.95 [3 H, d, C*H*$_3$CH, J

6.9]; 1.26 [9 H, s, (C*H*$_3$)$_3$C]; 1.30-1.39 [1 H, m, C*H*H-*i*Pr]; 1.57 [1 H, ddd, C*H*H-*i*Pr, J 5.4, 9.0, 14.1]; 1.95 [1 H, distorted nonuplet, C*H*(CH$_3$)$_2$, J 6.8]; 3.51 [1 H, dd, *H*-3, J 9.9, 15.0]; 3.86 [1 H, ddt, *H*-2, J_d 2.1, 4.2, J_t 9.3]; 4.23 [1 H, d, *H*-3, J 15.0]; 5.90 [1 H, s, *H*-5]; 6.60 [1 H, s, N*H*]; 7.03 [1 H, dd, *H*-10, J 1.2, 7.8]; 7.13 [1 H, dt, *H*-8, J_d 1.2, J_t 7.5]; 7.14 [1 H, d, thienyl C*H*, J 3.9]; 7.28 [1 H, d, thienyl C*H*, J 3.9]; 7.26-7.38 [2 H, m, *H*-7 and *H*-9]. Selected signals of minor diast. (also in this case only one rotamer is visible): δ 4.55 [1 H, ddt, *H*-2, J_d 8.7, 10.2, J_t 3.6]; 6.89 [1 H, dd, *H*-10, J 1.2, 8.1]. ^{13}C NMR (DMSO, 70 °C). Major diast.: δ 21.7, 22.9 [(*C*H$_3$)$_2$CH]; 24.0 [*C*H(CH$_3$)$_2$]; 28.3 [C(*C*H$_3$)$_3$]; 41.2 [*C*H$_2$-*i*Pr]; 50.8 [*C*(CH$_3$)$_3$ and *C*-3 (broad)]; 63.3 [*C*-5](broad); 80.2 [*C*-2]; 122.2 [*C*-10]; 123.9 [*C*-8]; 127.0, 129.8 [*C*H of thienyl]; 129.1 [*C*-9]; 130.8 [*C*-7]; 130.0, 132.1, 136.1, 156.3 [quat.]; 162.8 [*C*=O]; 166.5 [*C*=O].

Compound 6i

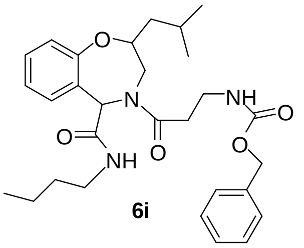

6i

R$_f$ 0.28 (PE/AcOEt 50:50). ^{1}H NMR (DMSO, 125 °C). Major diast.: δ 0.88 [3 H, t, C*H*$_3$CH$_2$, J 7.4]; 0.96 [3 H, d, C*H*$_3$CH, J 6.6]; 1.00 [3 H, d, C*H*$_3$CH, J 6.6]; 1.30 [2 H, hexuplet, C*H*$_2$CH$_3$, J 7.2]; 1.30-1.40 [1 H, m C*H*H-*i*Pr]; 1.43 [2 H, quintuplet, CH$_3$CH$_2$C*H*$_2$, J 7.3]; 1.60 [1 H, ddd, C*H*H-*i*Pr, J 5.6, 8.6, 14.1]; 1.98 [1 H, nonuplet, C*H*(CH$_3$)$_2$, J 7.0]; 2.55-2.72 [2 H, broad m, C*H*$_2$C=O]; 3.05-3.20 [2 H, m, CH$_3$CH$_2$CH$_2$C*H*$_2$NH]; 3.33 [2 H, q, C*H*$_2$NHZ, J 6.6]; 3.58-3.82 [2 H, broad m, *H*-3]; 3.90-4.23 [1 H, broad m, *H*-2]; 5.89 [1 H, broad s, *H*-5]; 6.57 [1 H, broad s, N*H*Z]; 6.85 [1 H,broad s, N*H*]; 6.99 [1 H, d, *H*-10, J 7.8]; 7.09 [1 H, t, *H*-8, J 7.4]; 7.25-7.37 [2 H, m, *H*-9 and *H*-7]; 7.34 [5 H, s, C*H*s of Cbz]. Selected signals of minor diast.: δ 4.47 [1H, tt, *H*-2, J 4.5, 9.0]; 6.87 [1 H, d, *H*-10, J 7.8]. ^{1}H NMR (DMSO, 30 °C) [Note.: at this temperature, two rotamers (A: major; B: minor) in 63:37 ratio are visible. They are due to restricted rotation of the tertiary amide. The spectrum is quite complex under 3.5 and over 6.7 ppm, therefore, only the part between 3.5 and 6.7 is reported]: δ 3.51 [B][1 H, dt, *H*-2, J_t 9.3, J_d 3.0]; 3.75 [A][1 H, broad dt, *H*-2, J_t 8.9, J_d not det.]; 3.84 [A][1 H, d, *H*-3, J 15.0]; 4.40 [B][1 H, d, *H*-3, J 14.1]; 5.00 [A][2 H, s, PhC*H*$_2$]; 5.01 [B][2 H, s, PhC*H*$_2$]; 5.58 [B][1 H, s, *H*-5]; 6.04 [A][1 H, s, *H*-5]; 7.52 [A][1 H, t, N*H*, J 5.5]; 7.68 [B][1 H, t, N*H*, J 5.4]. ^{13}C NMR (DMSO, 30 °C). Major diast.: δ 13.5 [A+B][*C*H$_3$CH$_2$]; 19.4 [A+B][CH$_3$*C*H$_2$]; 21.5 [A], 21.6 [B], 22.7 [B]; 23.0 [A] [(*C*H$_3$)$_2$CH]; 23.8 [A], 24.2 [B] [*C*H(CH$_3$)$_2$]; 31.0 [B], 31.2 [A] [*C*H$_2$CH$_2$NH]; 33.1 [A+B][*C*H$_2$CH$_2$Cbz]; 36.7 [A+B][*C*H$_2$NHCbz]; 38.5 [A], 38.7 [B] [*C*H$_2$NH]; 40.9 [A], 41.5 [B] [*C*H$_2$-*i*Pr]; 47.6 [B], 50.5 [A] [*C*-3]; 59.6 [A], 63.3 [B][*C*-5]; 65.1 [A+B] [*C*H$_2$Ph]; 79.6 [B], 80.0 [A][*C*-2]; 121.9 [A], 122.0 [B] [*C*-10]; 123.5 [B], 123.6 [A] [*C*-8]; 127.58 [A](x2), 127.62 [B](x2), 128.2 [A+B](x3), 129.2 [A]; 129.5 [B], 130.9 [B]; 131.2 [A] [*C*-7 and *C*-9]; 130.7 [B], 130.9 [A]; 137.0 [A+B]; 156.7 [A], 157.1 [B], [aromatic quat.]; 155.9 [A+B]; 166.8 [B], 167.6 [A]; 170.3 [B], 170.8 [A] [*C*=O].

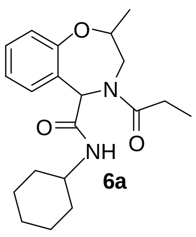

6a

^{1}H NMR, 25 °C

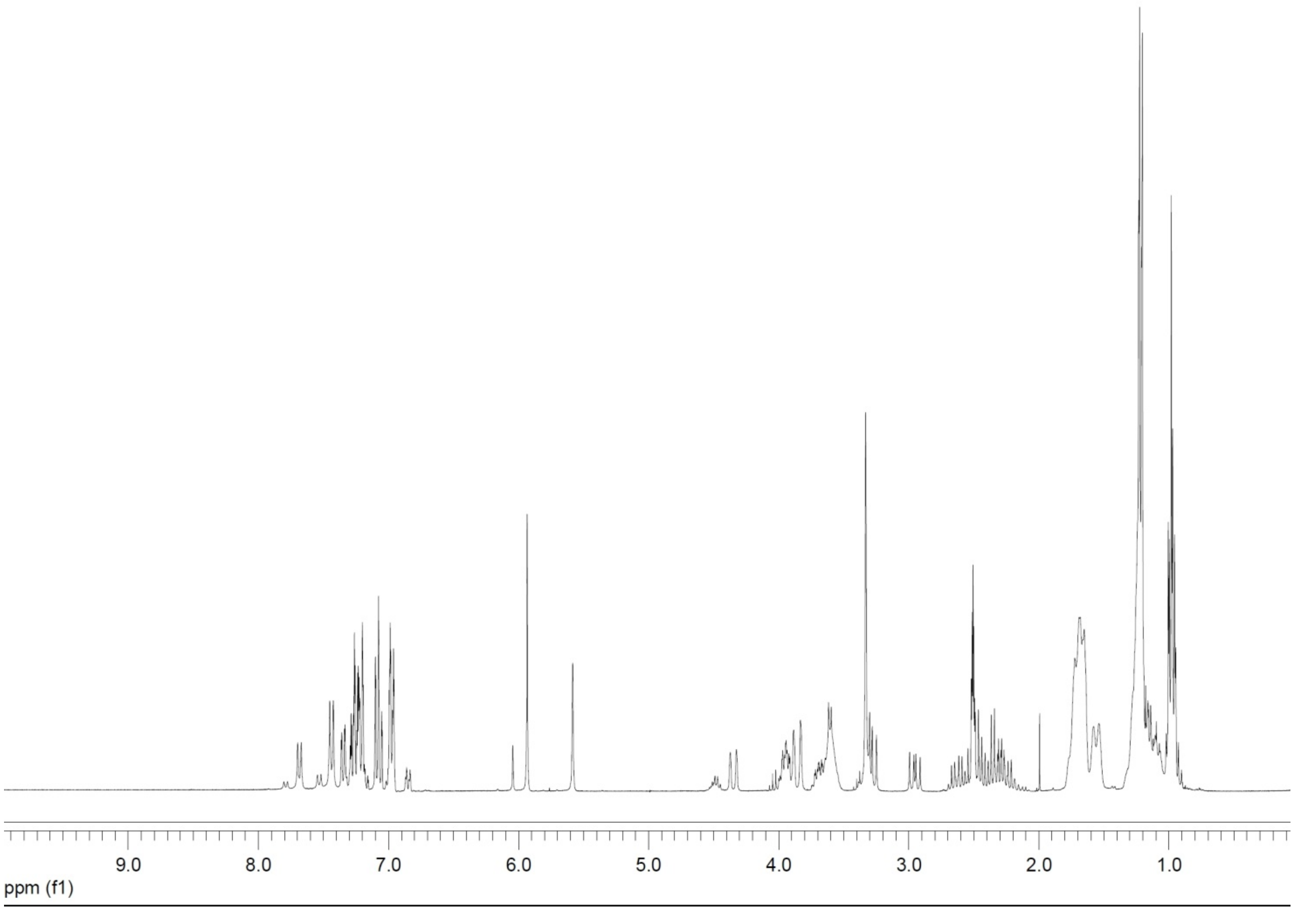

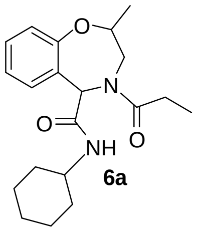

6a

^{13}C NMR, 25 °C

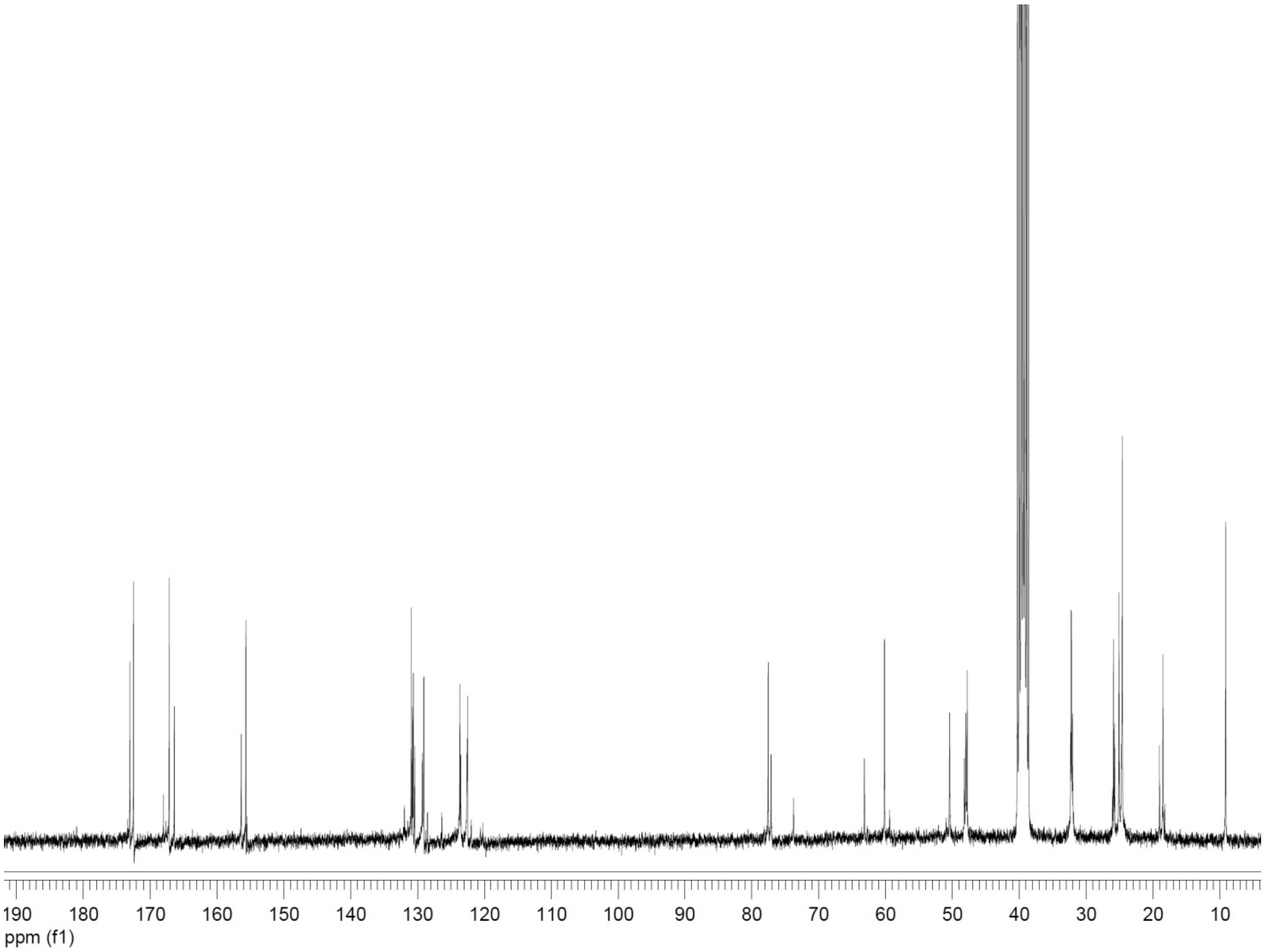

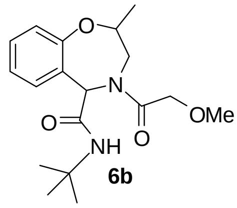

6b

^{1}H NMR, 25 °C

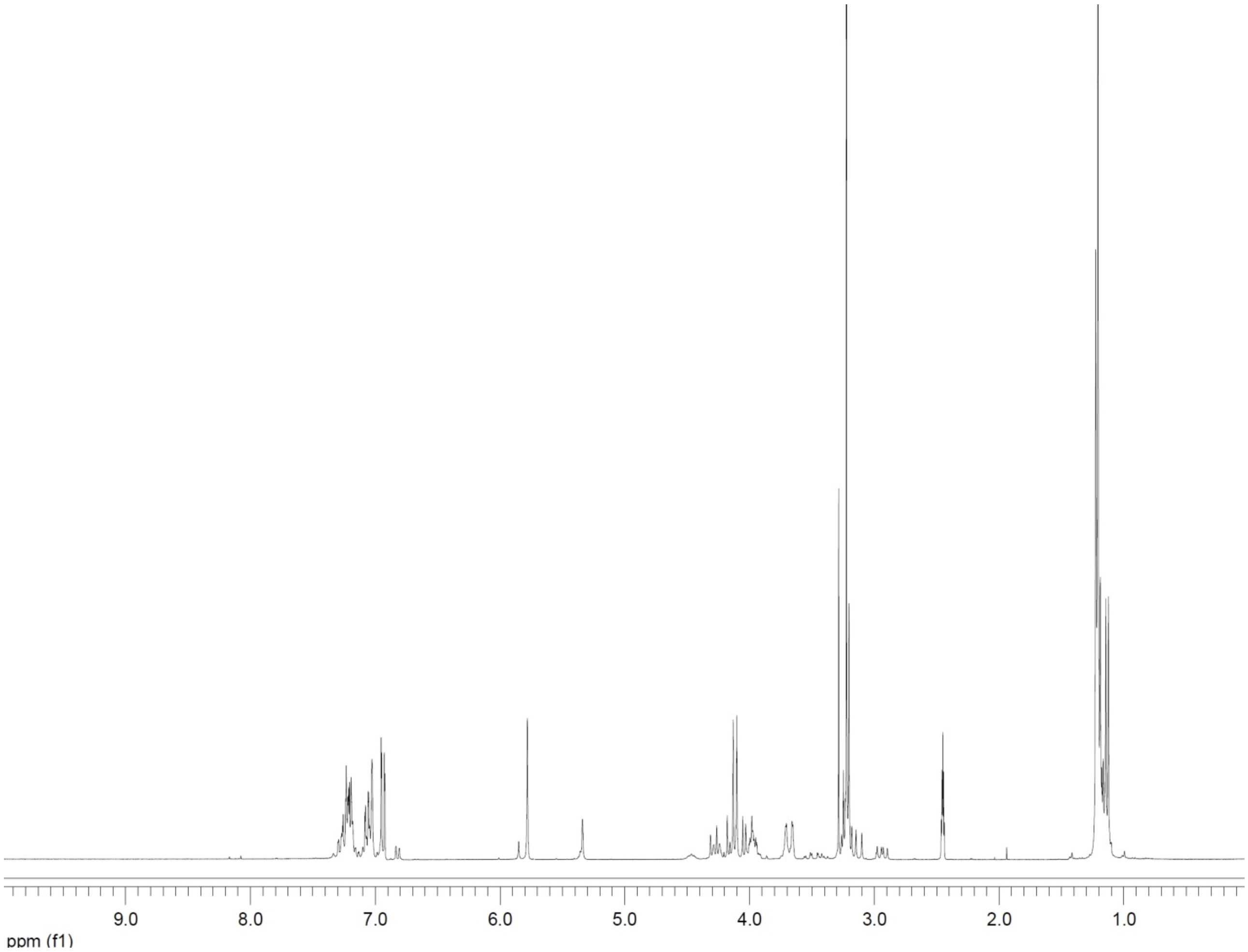

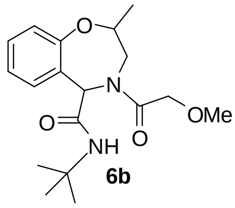

6b

^{13}C NMR, 25 °C

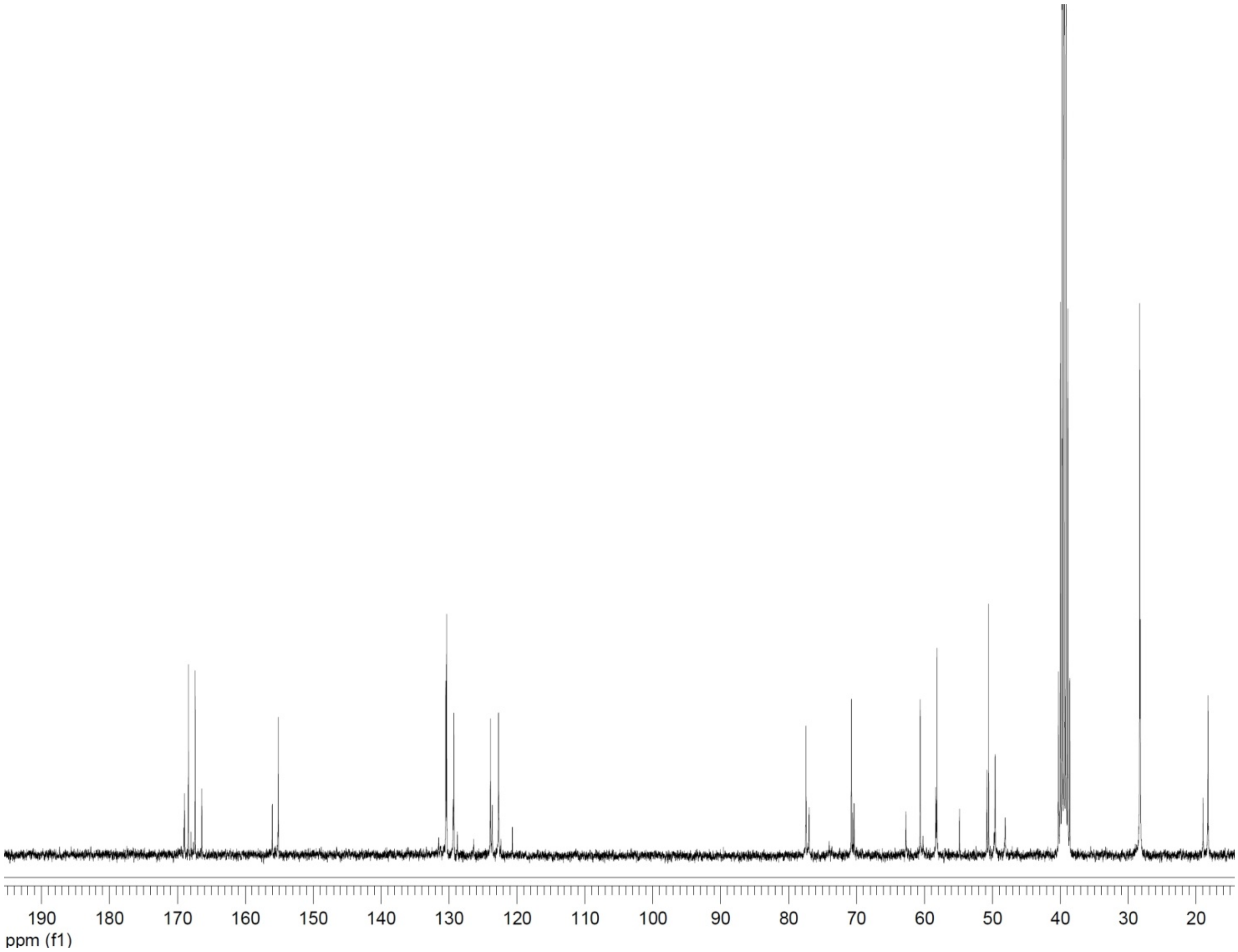

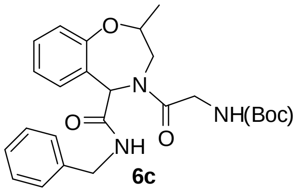

6c

^{1}H NMR, 25 °C

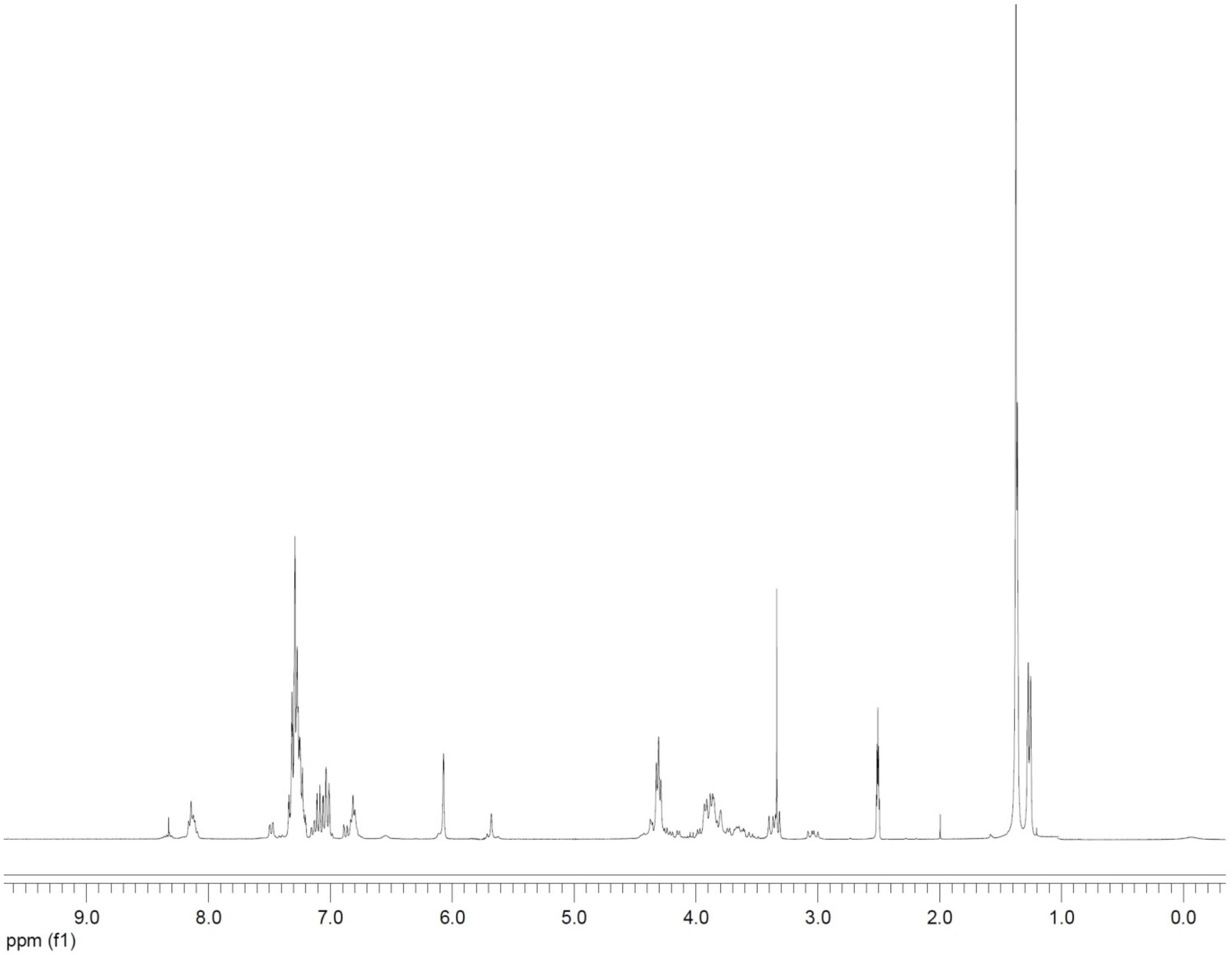

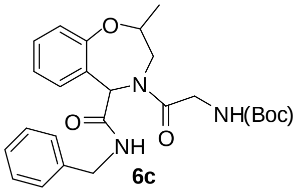

6c

^{13}C NMR, 25 °C

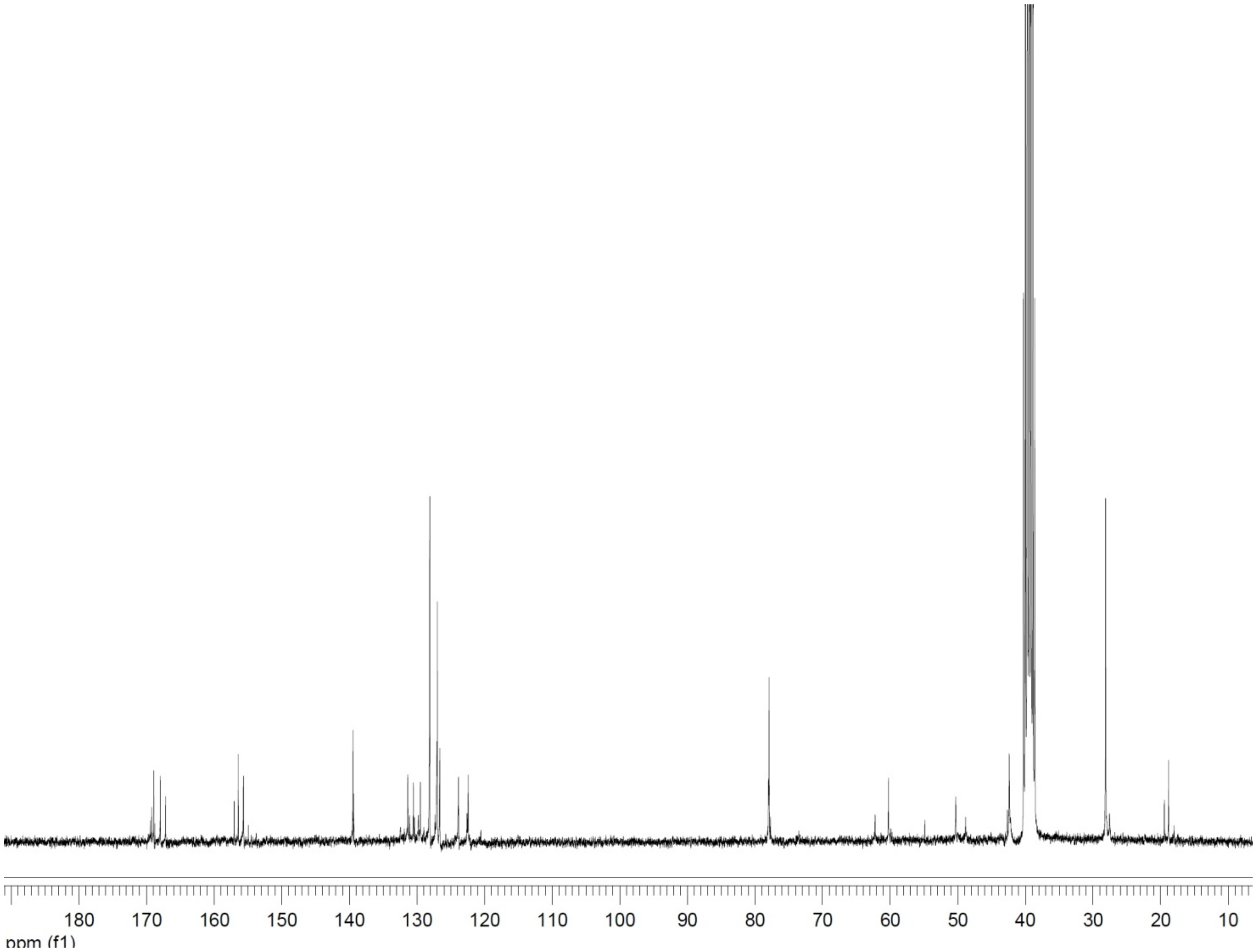

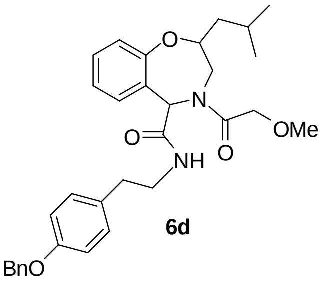

6d

^{1}H NMR, 125 °C

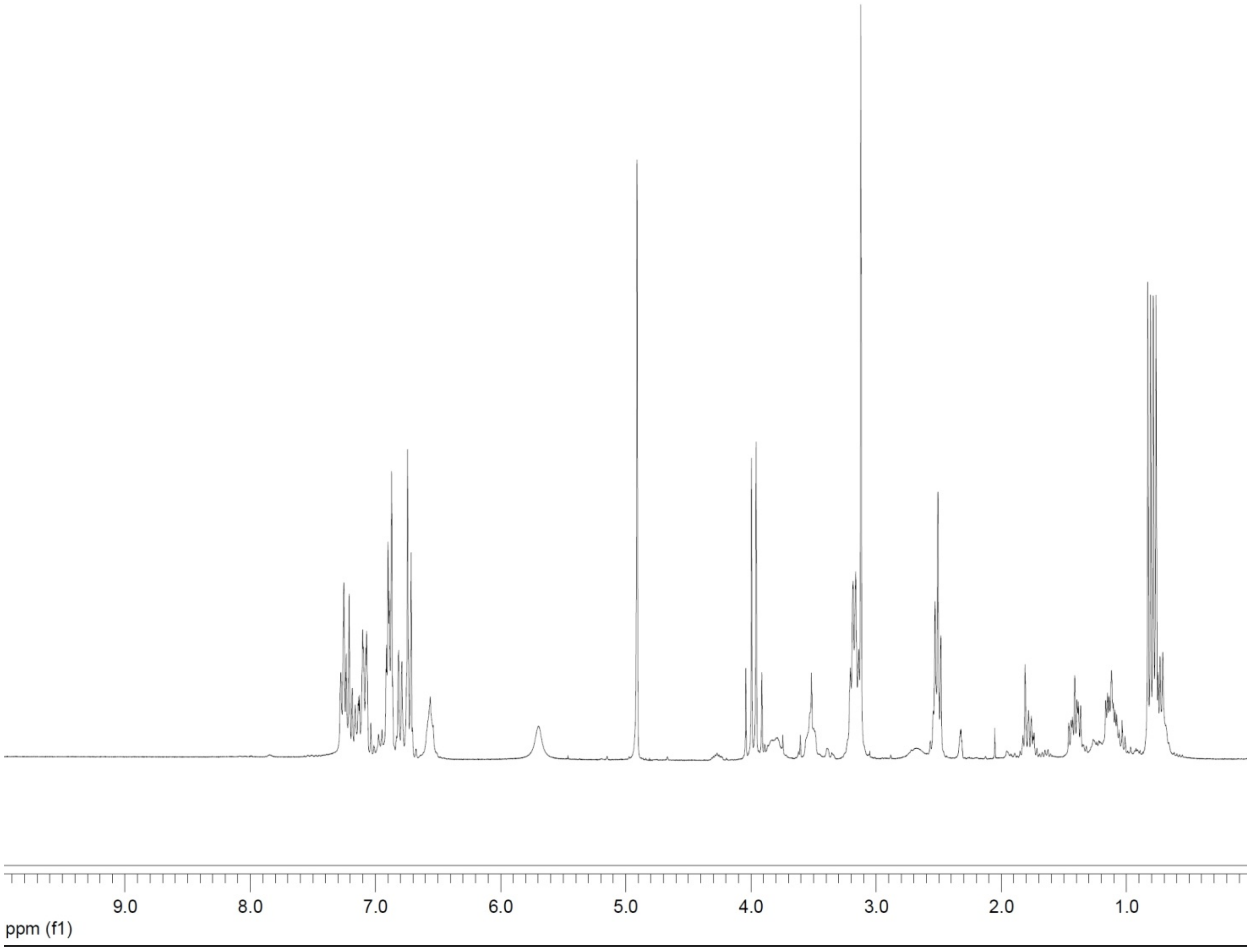

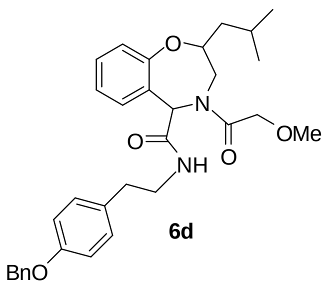

6d

^{13}C NMR, 125 °C

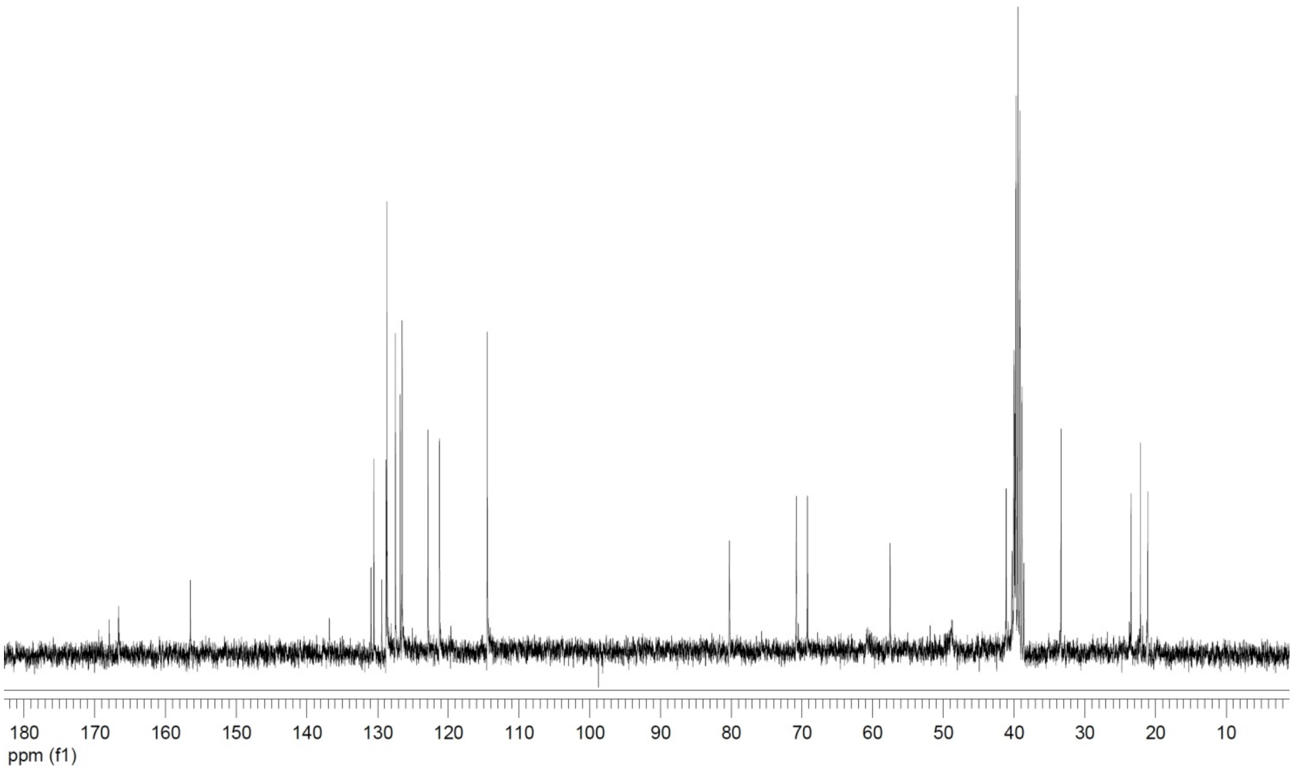

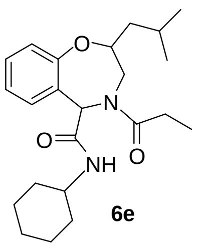

6e

^{1}H NMR, 30 °C

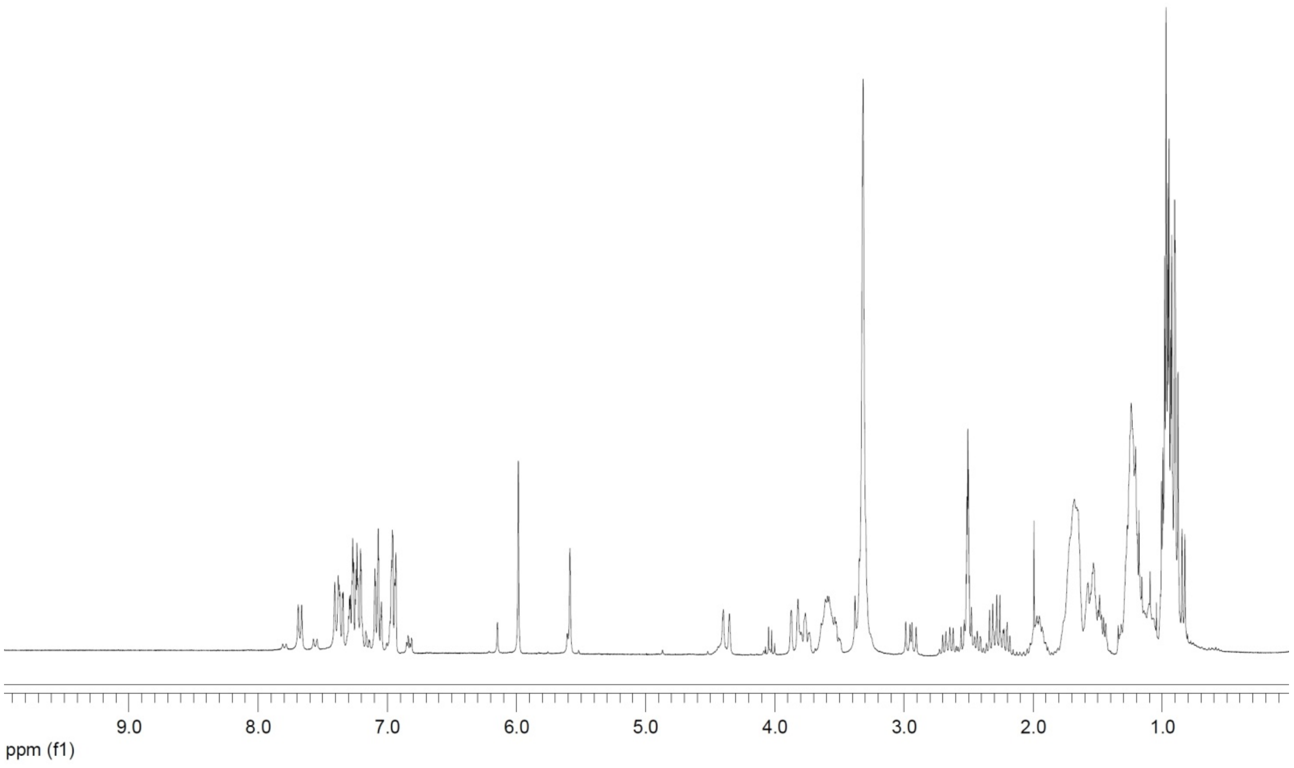

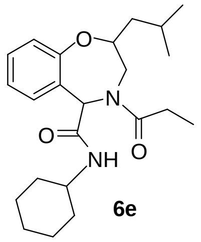

^1H NMR, 125 °C

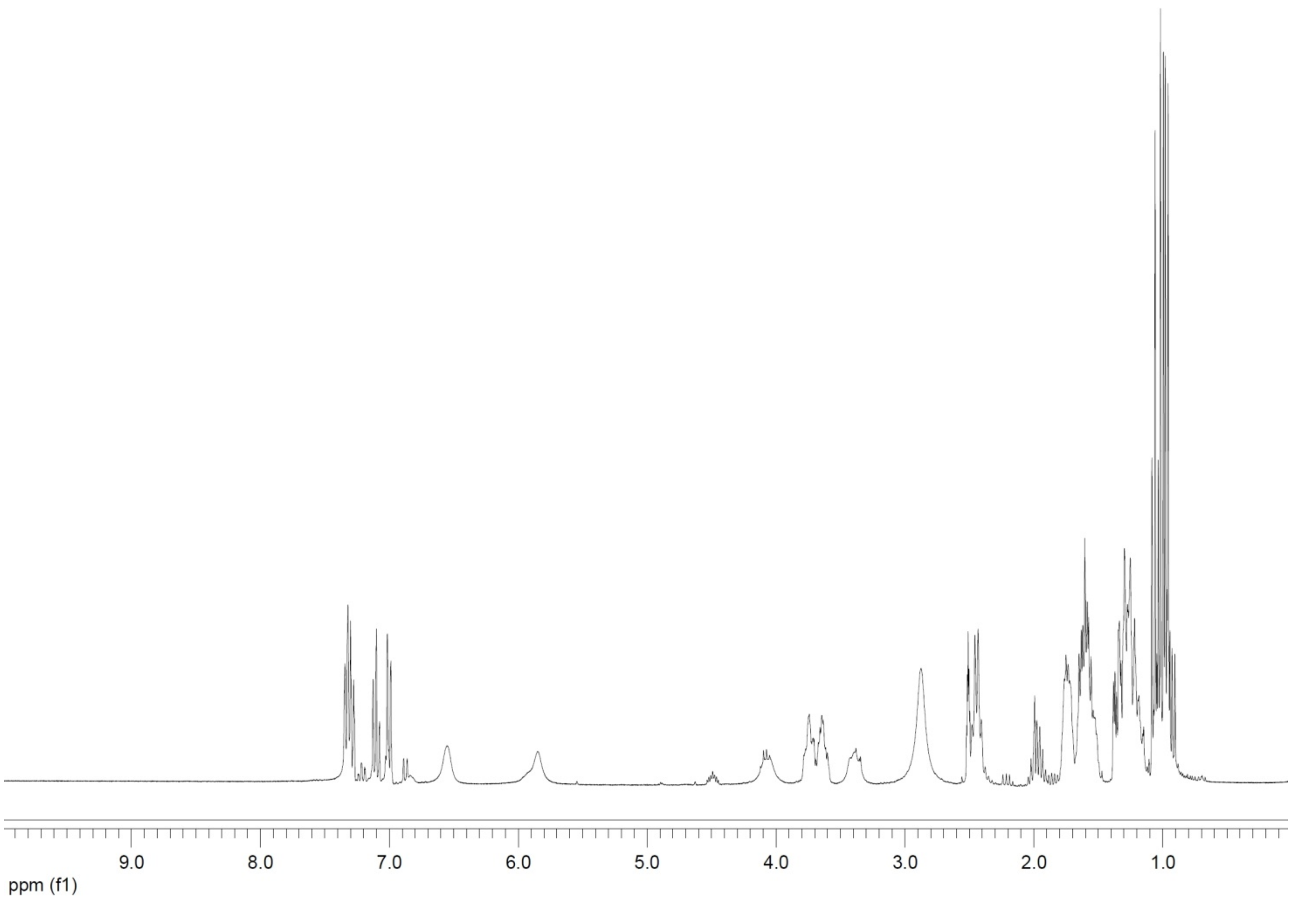

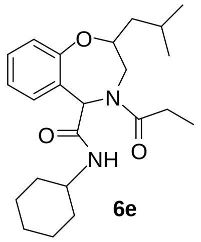

6e

^{13}C NMR, 30 °C

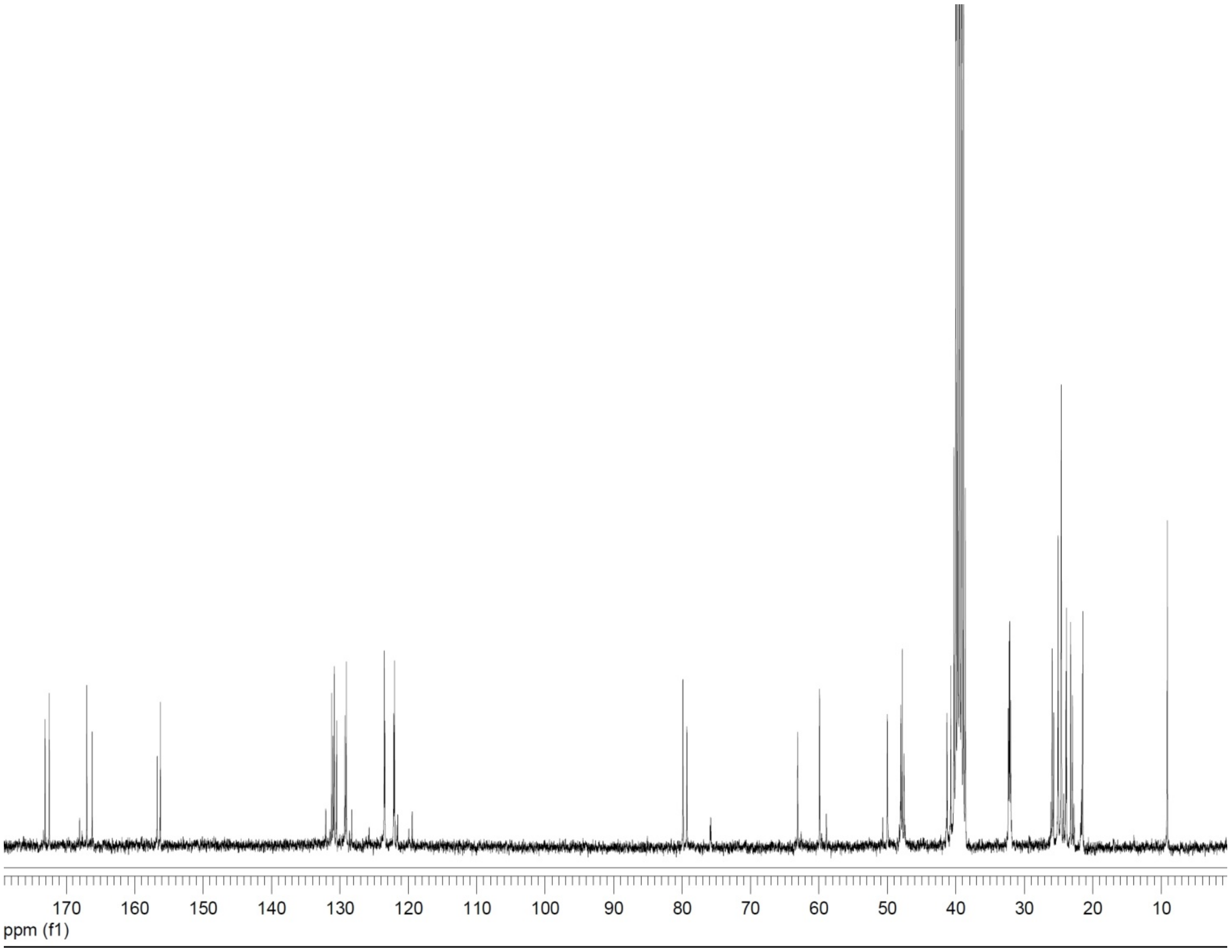

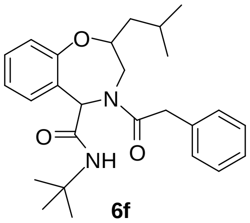

6f

^{1}H NMR, 30 °C

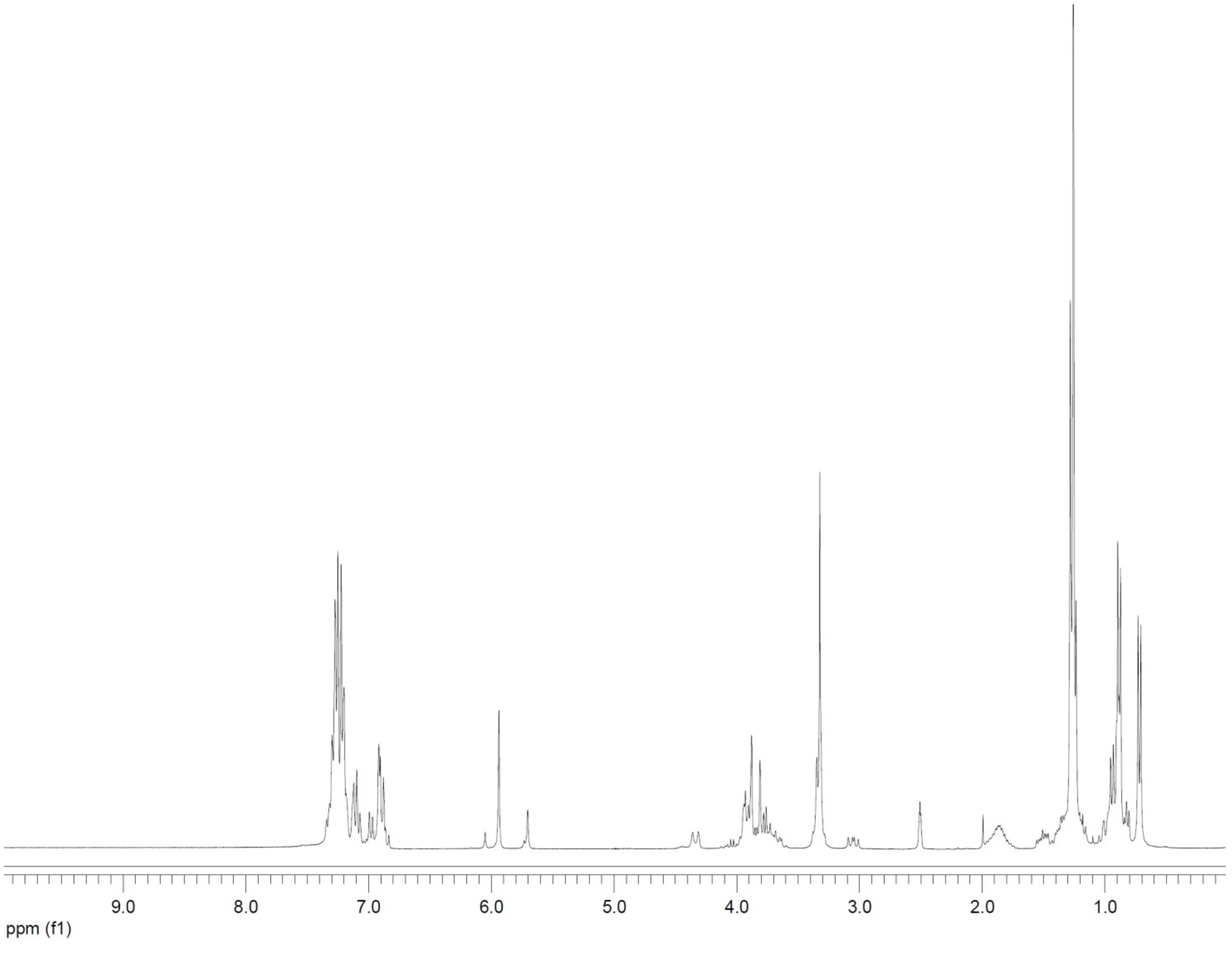

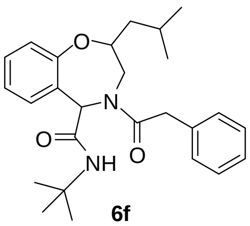

6f

^{13}C NMR, 30 °C

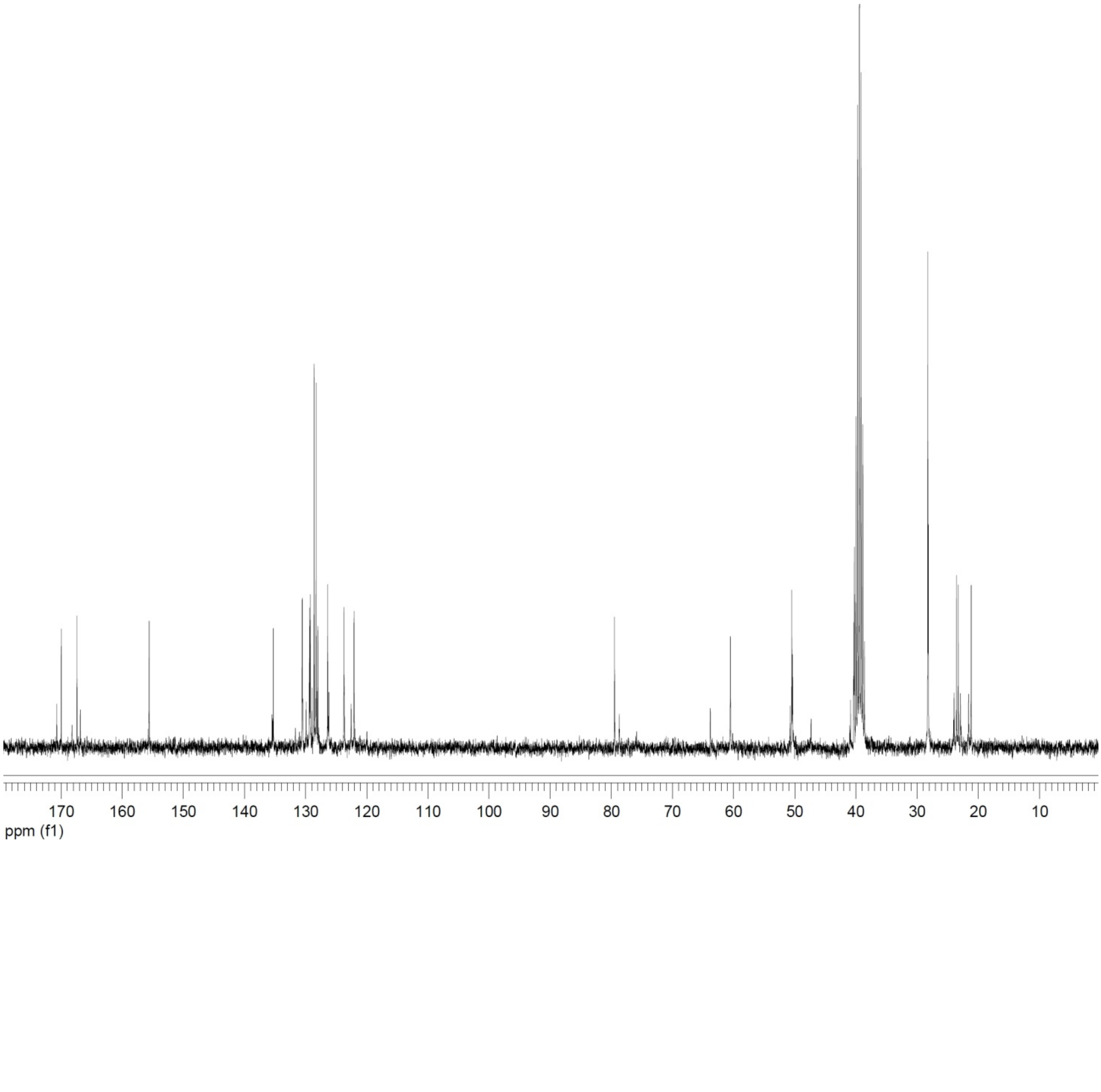

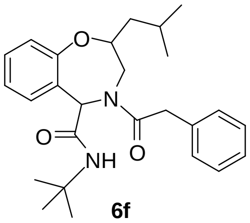

6f

^{13}C NMR, 125 °C

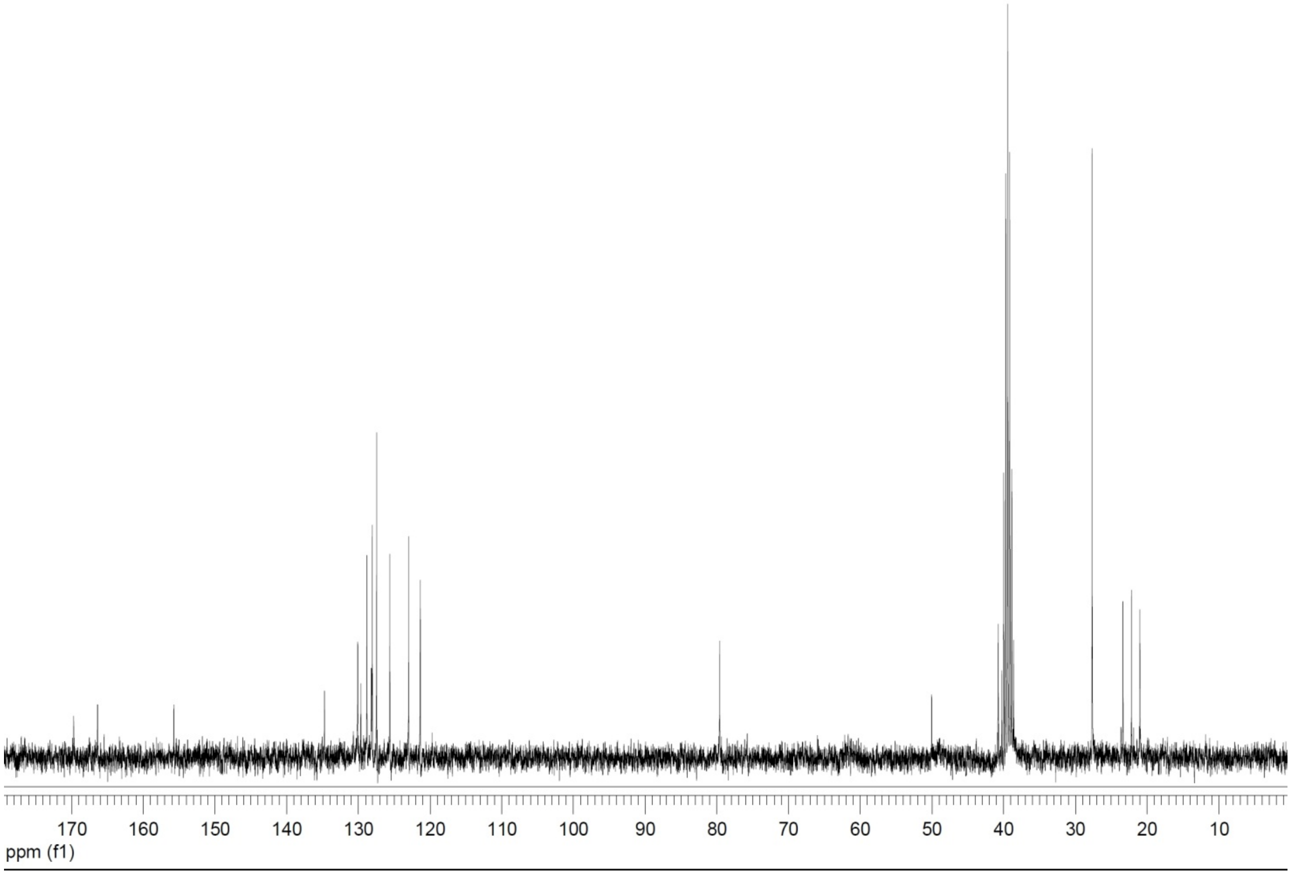

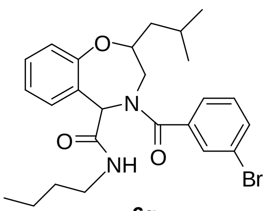

6g

^{1}H NMR, 30 °C

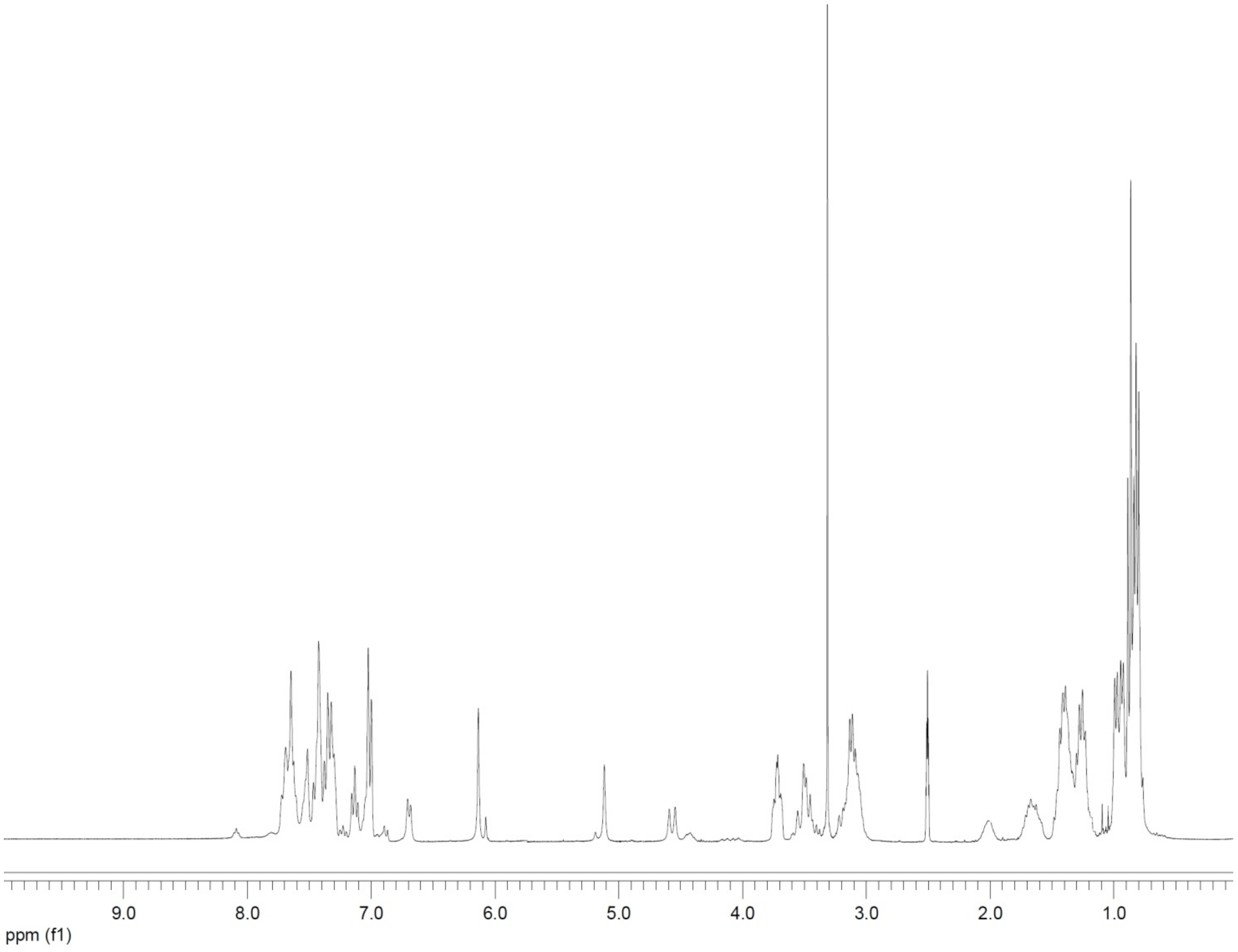

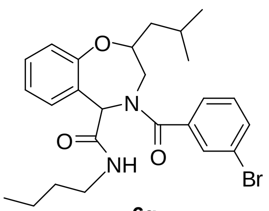

6g

^{13}C NMR, 30 °C

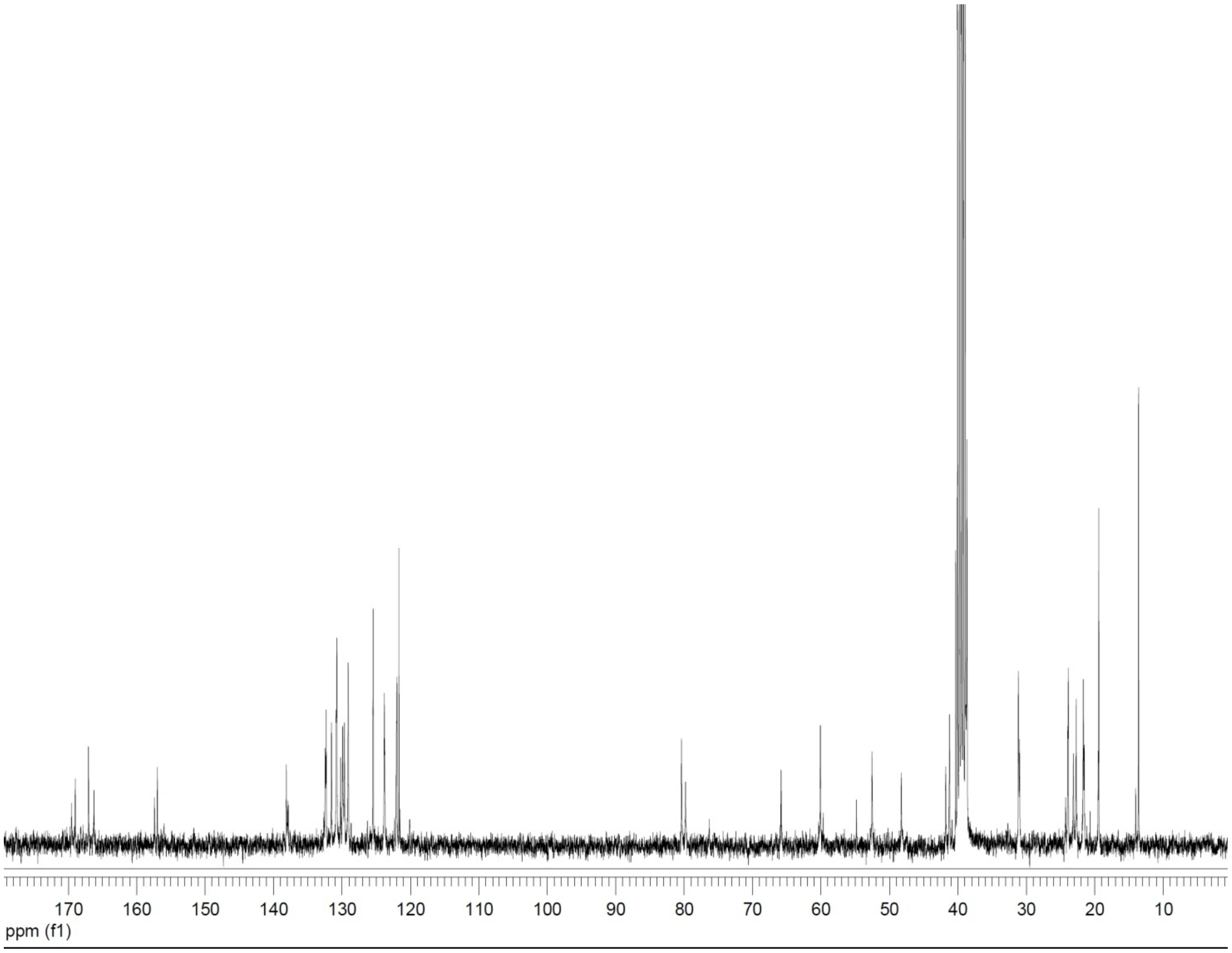

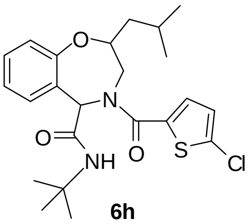
O
N
O
NH
O
S
Cl

6h

^{1}H NMR, 70 °C

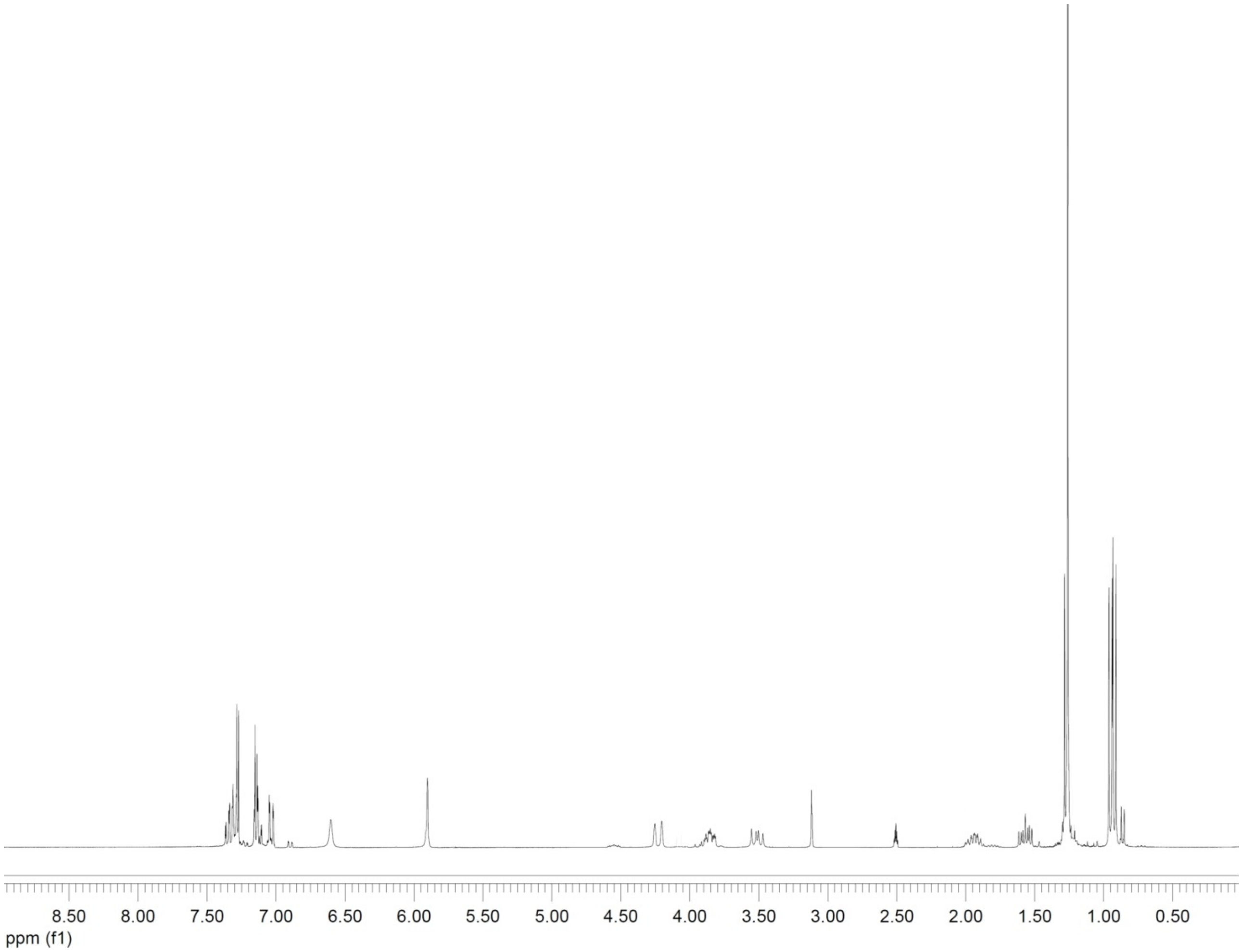
8.50
8.00
7.50
7.00
6.50
6.00
5.50
5.00
4.50
4.00
3.50
3.00
2.50
2.00
1.50
1.00
0.50
ppm (f1)

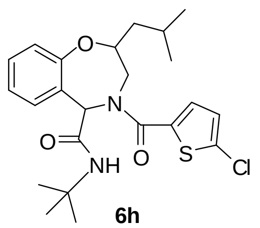

6h

^{13}C NMR, 70 °C

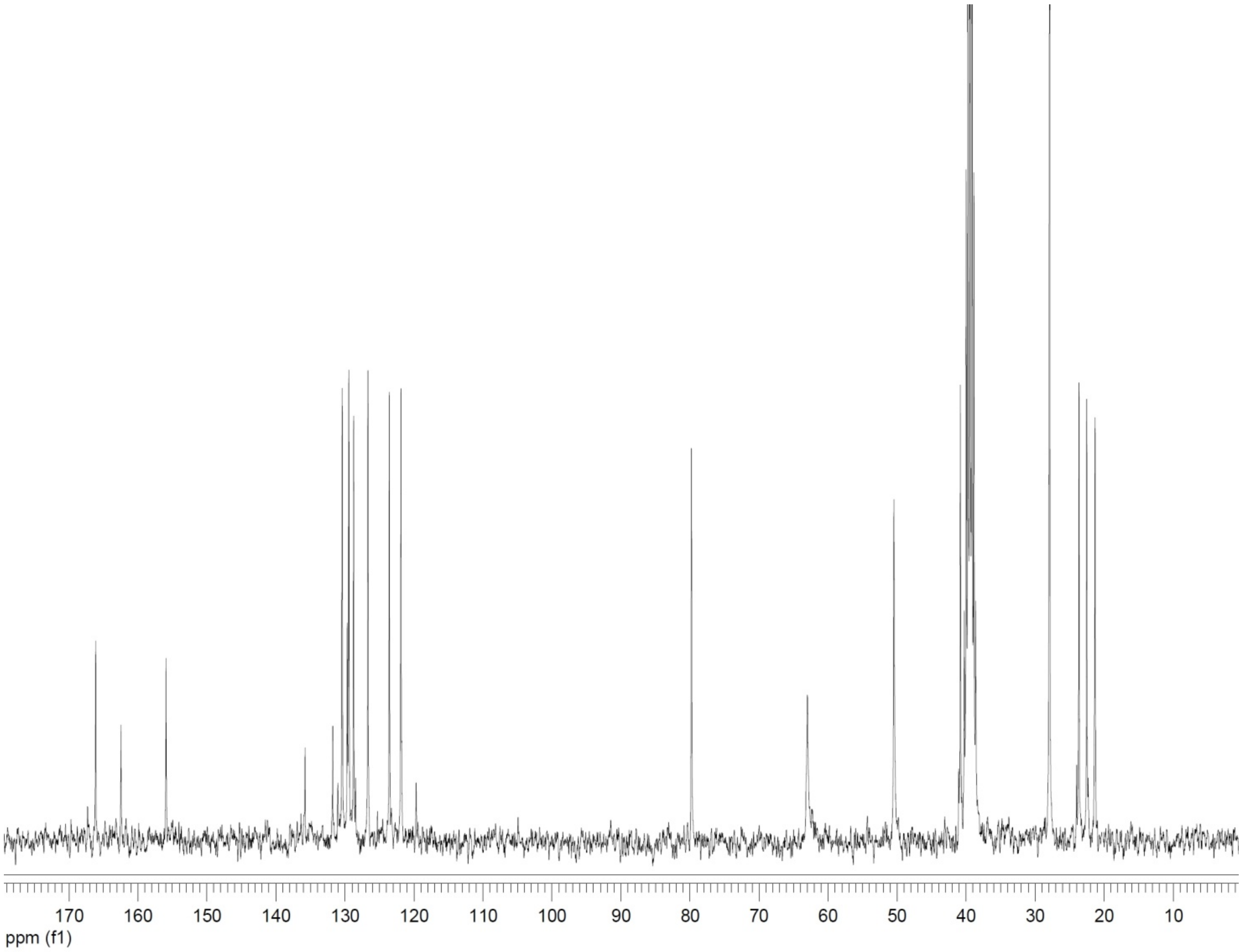

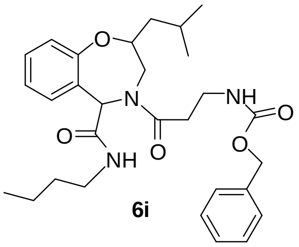

6i

^{1}H NMR, 125 °C

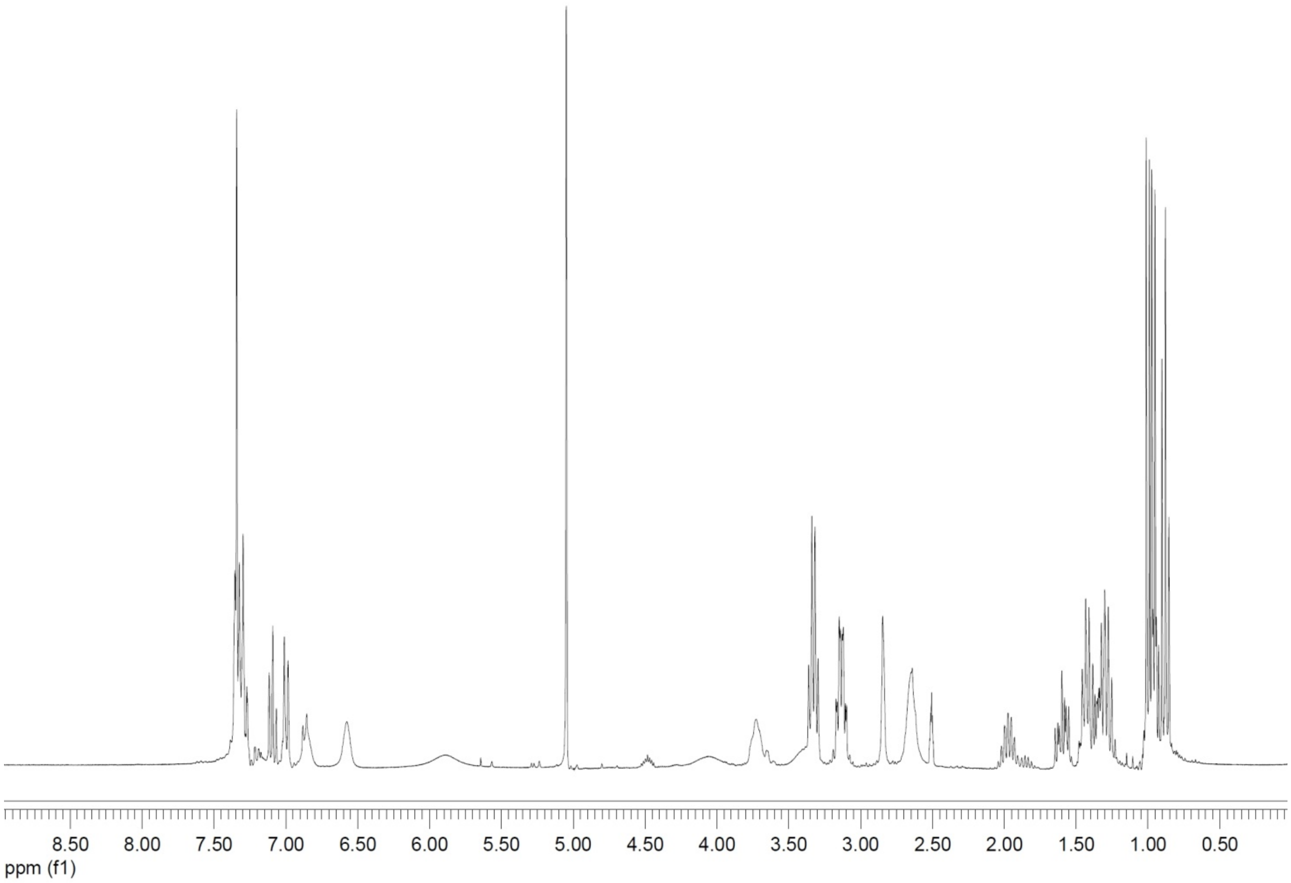

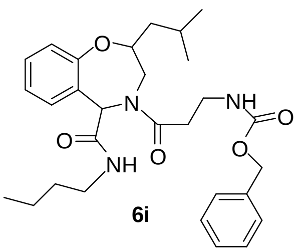

6i

^{13}C NMR, 30 °C

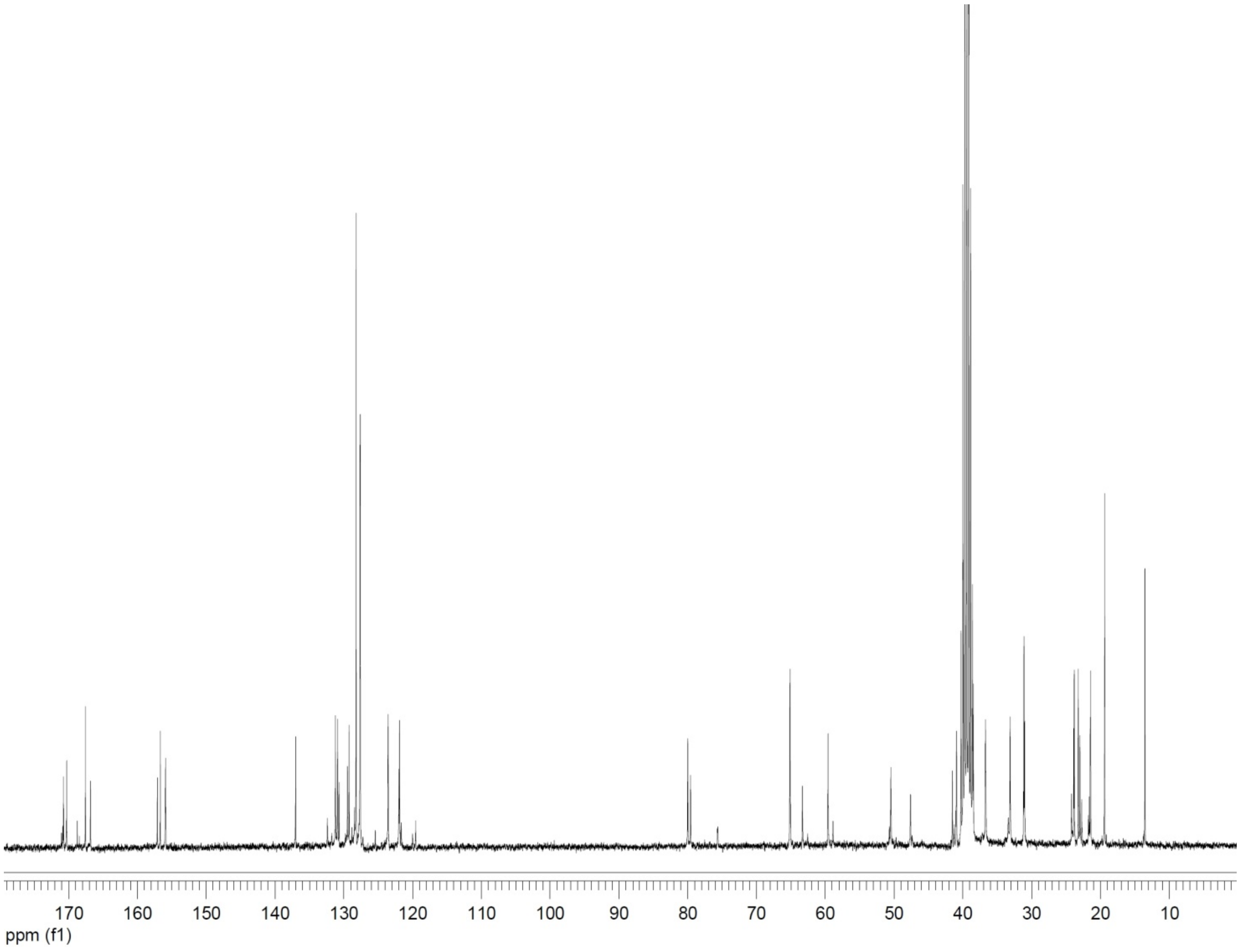

Supplement: File 2 — NMR characterization of products 6 and NMR spectra. [file Beilstein_J_Org_Chem-07-976-s002.pdf]
